# Site-specific chromosomal gene insertion: Flp recombinase *versus* Cas9 nuclease

**DOI:** 10.1038/s41598-017-17651-0

**Published:** 2017-12-19

**Authors:** Quang Vinh Phan, Jörg Contzen, Petra Seemann, Manfred Gossen

**Affiliations:** 1 Berlin-Brandenburg Center for Regenerative Therapies (BCRT), Föhrer Strasse 15, 13353 Berlin, Germany; 2 Charité - Universitätsmedizin Berlin, Augustenburger Platz 1, 13353 Berlin, Germany; 3 Helmholtz-Zentrum Geesthacht (HZG), Institute of Biomaterial Science, Teltow, 14513 Germany; 4 Present Address: Charité - Universitätsmedizin Berlin, Labor für Pädiatrische Molekularbiologie, Berlin, Germany

## Abstract

Site-specific recombination systems like those based on the Flp recombinase proved themselves as efficient tools for cell line engineering. The recent emergence of designer nucleases, especially RNA guided endonucleases like Cas9, has considerably broadened the available toolbox for applications like targeted transgene insertions. Here we established a recombinase-mediated cassette exchange (RMCE) protocol for the fast and effective, drug-free isolation of recombinant cells. Distinct fluorescent protein patterns identified the recombination status of individual cells. In derivatives of a CHO master cell line the expression of the introduced transgene of interest could be dramatically increased almost 20-fold by subsequent deletion of the fluorescent protein gene that provided the initial isolation principle. The same master cell line was employed in a comparative analysis using CRISPR/Cas9 for transgene integration in identical loci. Even though the overall targeting efficacy was comparable, multi-loci targeting was considerably more effective for Cas9-mediated transgene insertion when compared to RMCE. While Cas9 is inherently more flexible, our results also alert to the risk of aberrant recombination events around the cut site. Together, this study points at the individual strengths in performance of both systems and provides guidance for their appropriate use.

## Introduction

The accurate, site-specific genetic manipulation of mammalian chromosomes remains a technical challenge. This holds true for targeted insertions, deletions or substitutions of DNA that can range in size from entire chromosomal regions to individual nucleotides. The challenges include limiting genome alterations exclusively to the target site, to obtain reasonably high targeting efficacy in a reliable manner, and to successfully select engineered cells. Homologous recombination solely based on extended sequence identity between the exogenous donor and the endogenous acceptor locus is widely employed. However, this strategy is restricted to selected cell types like certain mouse embryonic stem cells^1^. In contrast, site-specific targeting based on recombinases like Cre, Flp or Phi (see reference^2^ for a recent review) has been used for decades without apparent cell type preferences. While these recombinase systems are widely employed for cell line engineering, they inevitably require the prior establishment of master cell lines genetically engineered to contain suitably arranged recombinase recognition sites as chromosomal targets, also referred to as “landing pads”. Such master cell lines are the starting point for the construction of isogenic cell lines, which are genetically almost identical aside from small, defined alterations, comprising the genetic element of interest. This can, for example, be an expression control sequences like a promoter or a gene of interest (GOI). For the analysis of regulatory genetic elements the main conceptual advantage for the use of recombinase-mediated targeting to predetermined loci is to minimize integration site effects on the experimental readout between different isogenic lines^3–5^. Such genetic elements are never truly autonomous and subject to proximal chromatin structures as well as regulatory signals located in *cis*. However, comparative analyses in isogenic lines would impose the same external influences on all the regulatory elements to be analysed. In contrast, the repeated insertion of different protein-encoding genes in the same, pre-characterised chromosomal site is frequently applied in biotechnological research, including the establishment of production cell lines for biologics. Here, the main rational behind this strategy is that a high producer line, once identified in an often tedious and time consuming process, can be re-utilized for the production of another gene product, while the favourable characteristics of the original cell line are preserved^6–9^. Such recombinase-mediated, site-specific insertion of DNA fragments in mammalian chromosomes can be achieved by integration of the entire donor vectors (DV)^10,11^ or by cassette exchange methods like RMCE^12^). The latter process involves the substitution of segments of the chromosomally integrated landing pad-containing vector (LPV) by matching DV segments. This reaction requires only short recombinase recognition sites engineered properly in both, the target region on the chromosome and the incoming DV.

Several of the engineering strategies outlined so far can also be realized using the more recently developed designer nucleases^13–15^. Their use for site-specific chromosomal DNA insertion provides greater flexibility in target site selection when compared to a recombinase strategy. DNA double-strand cuts by these nucleases close to the intended insertion site facilitate a homology-driven repair (HDR) mechanism, provided that a properly designed DV that contains the insert flanked by homology arms identical to the chromosomal sequences around the actual cut site is present^16^. Such alternative strategies, especially the use of RNA-guided engineered nucleases (RGENs) like in Clustered Regularly Interspaced Short Palindromic Repeats/CRISPR-associated protein-9 nuclease (CRISPR/Cas9) genome editing, can substantially speed up targeted insertions when no premade master cell lines are available.

Here we established a RMCE protocol in which recombination events can be monitored and isolated by following expression of fluorescent marker proteins. This procedure proved highly efficient to obtain cell lines with homogenous, phenotypically stable transgene expression. A side-by-side comparison to CRISPR/Cas9-mediated targeting of the same chromosomal loci showed comparable efficiencies of donor vector integration. However, when two potential integration sites were present in a given cell, striking differences between the recombinase- and the nuclease-based genome engineering systems were observed, with CRISPR/Cas9 having a much higher capacity to simultaneously modify multiple loci at a time.

## Results

### Targeted Chromosomal Transgene Integration: Vector Design Rationales

**Figure 1:**
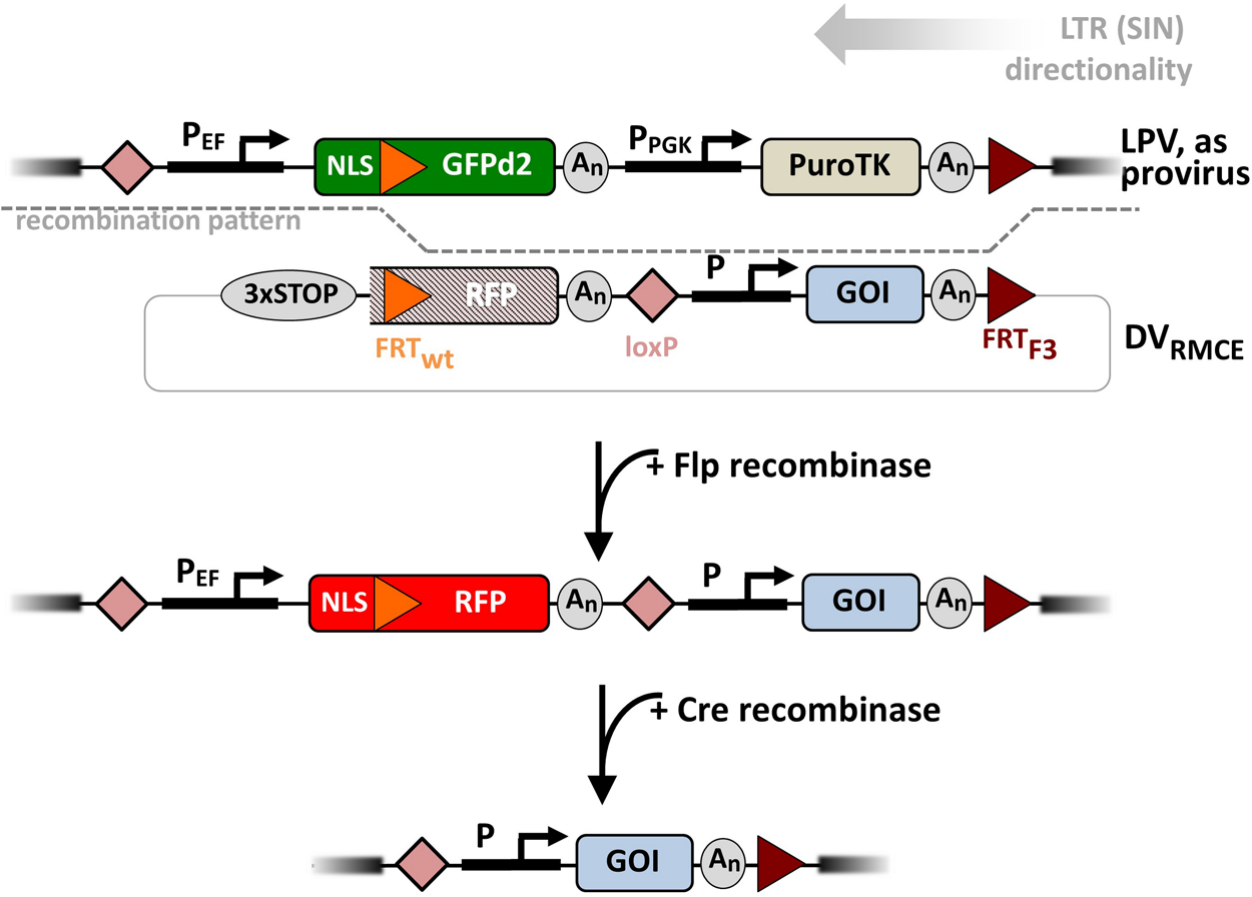
RMCE strategy. The landing pad vector (LPV) including the GFP reporter and the PuroTK selection marker gene is integrated into the host genome by lentiviral transduction. The donor vector (DV_RMCE_) includes a non-functional RFP reporter and the GOI expression unit (upper panel). Flp recombinase-mediated cassette exchange of the FRT-flanked DV_RMCE_ sequences results in the functional reconstitution of the RFP reporter gene along with the insertion of the GOI (middle panel). If desired, the fluorescent reporter can be selectively deleted via Cre recombination (lower panel). See *Results* section for vector features and abbreviations.

As a general strategy to monitor site-specific donor integration in a preselected chromosomal target, the landing pad, we chose to follow the expression of two spectral distinct fluorescent proteins. The favourable properties of stable cell lines isolated via cell sorting according to fluorescent protein gene expression are well established^17,18^. Here, the switch was from the landing pad vector (LPV)-encoded green fluorescent protein (GFP) to a red fluorescent protein (RFP). For Flp-mediated RMCE outlined in Fig. 1, top, two heterospecific Flp recombination targets (FRT sites)^12^ were included in the LPV. A FRT_wt_ site was placed 5′ within the GFP open reading frame and a FRT_F3_ 3′ to the LPV transcription units. Corresponding FRT sites in the donor vector for RMCE (DV_RMCE_) were placed 5′ to the truncated RFP coding sequence lacking a promoter and a translation start codon (FRT_wt_) and 3′ to the GOI (FRT_F3_), respectively. This design allowed to distinguish between targeted and random chromosomal integration of the incoming RFP-derived marker construct. Functional RFP expression will only occur after recruitment of the ATG start codon of LPV’s GFP gene via in-frame targeted integration(i. e. an ATG and promoter trap^19^).

Several other features to enhance the sensitivity, versatility and utility of the matched RMCE vector combination were included in the design. *(i)* The expression of the GFP gene within the LPV is controlled by the human elongation factor 1 alpha (hEF1α) promoter. We have previously shown that this promoter favours high level, homogenous transgene expression, especially long term, in the absence of antibiotic selection^18,20^. *(ii)* A N-terminal nuclear localisation signal (NLS) is located 5′ to the GFP-embedded FRT_wt_. Nuclear accumulation of the fluorescent protein makes it easier to recognize low GFP levels against the cellular auto-fluorescence background^21^. This subcellular localisation feature will also pass on to the incoming RFP construct. This allows to stringently assess if a given red fluorescence signal observed originates from a targeted integration event (i.e. nuclear signal) or from the accidental acquisition of transcription/translation start signals via random integration, which in all likelihood would result in a non-localized signal. *(iii)* A GFP version with a C-terminal degradation domain (d2EGFP; d2 refers to a protein half-life of 2 hours)^22^ will result in faster GFP signal depletion after a DV_RMCE_ integration event. *(iv)* A PGK promoter driven positive/negative selection marker (+/−, here a puromycin/thymidine kinase gene – PuroTK)^23^ is included in the LPV, in case preparative fluorescence activated cell sorting (FACS) for exchange events will not be possible or practical. TK gene expression conveys sensitivity to Gancilovir. Thus, addition of this nucleoside analogue can be employed to monitor the absence of the PuroTK gene expected upon RMCE, eliminating site-specific insertion events of the whole DV_RMCE_ plasmid, which could in principle occur via recombinase action on FRT_wt_ alone^11^, that would result in GFP^−^/RFP^+^/PuroTK^+^ cells, or, in case integration via FRT_F3_ only, in GFP^+^/RFP^−^/PuroTK^+^ cells. *(v)* LoxP sites in both the LPV and DV are positioned such that the newly created RFP transcription unit, which is created upon successful RMCE, can be deleted via Cre-mediated excision^24^. *(vi)* Stop codons that immediately precede the DV_RMCE_’s FRT_wt_ site are positioned in all three potential reading frames^25^, to further preclude accidental RFP expression upon random integration in a promoter/ATG-trap constellation^19^. *(vii)* The DV_RMCE_ also contains a transcription unit for the actual gene-of-interest (GOI) for the respective study, which in the standard configuration is driven by the hEF1α promoter for the reasons outlined above^18,20^. *(viii)* Lastly, the LPV transcription units are integrated in a self-inactivating lentiviral backbone in opposite orientation to the LTR-directed transcription. Recombinant lentiviral particles not only facilitate highly efficient transduction of most mammalian cell lines, they also allow a fair command over integration copy number. In addition, the reverse orientation permits inclusion of unidirectional polyadenylation signals as well as an intron-containing hEF1α promoter in the virus, without premature termination or internal truncations of the viral genomic RNA. The resulting drop in viral titer when compared to LTR codirectionality^26^ was substantial, but manageable in our experiments.

The principle functionality of the proposed recombination scheme was initially established in an all-transient transfection experiment (i.e. co-transfection of LPV, DV_RMCE_ and Flp expression vectors). Only upon Flp expression red fluorescent cells emerged, indicative of successful recombination between LPV and DV_RMCE_ (suppl. Fig. S1).

### Characterisation of CHO RMCE master clones

**Figure 2:**
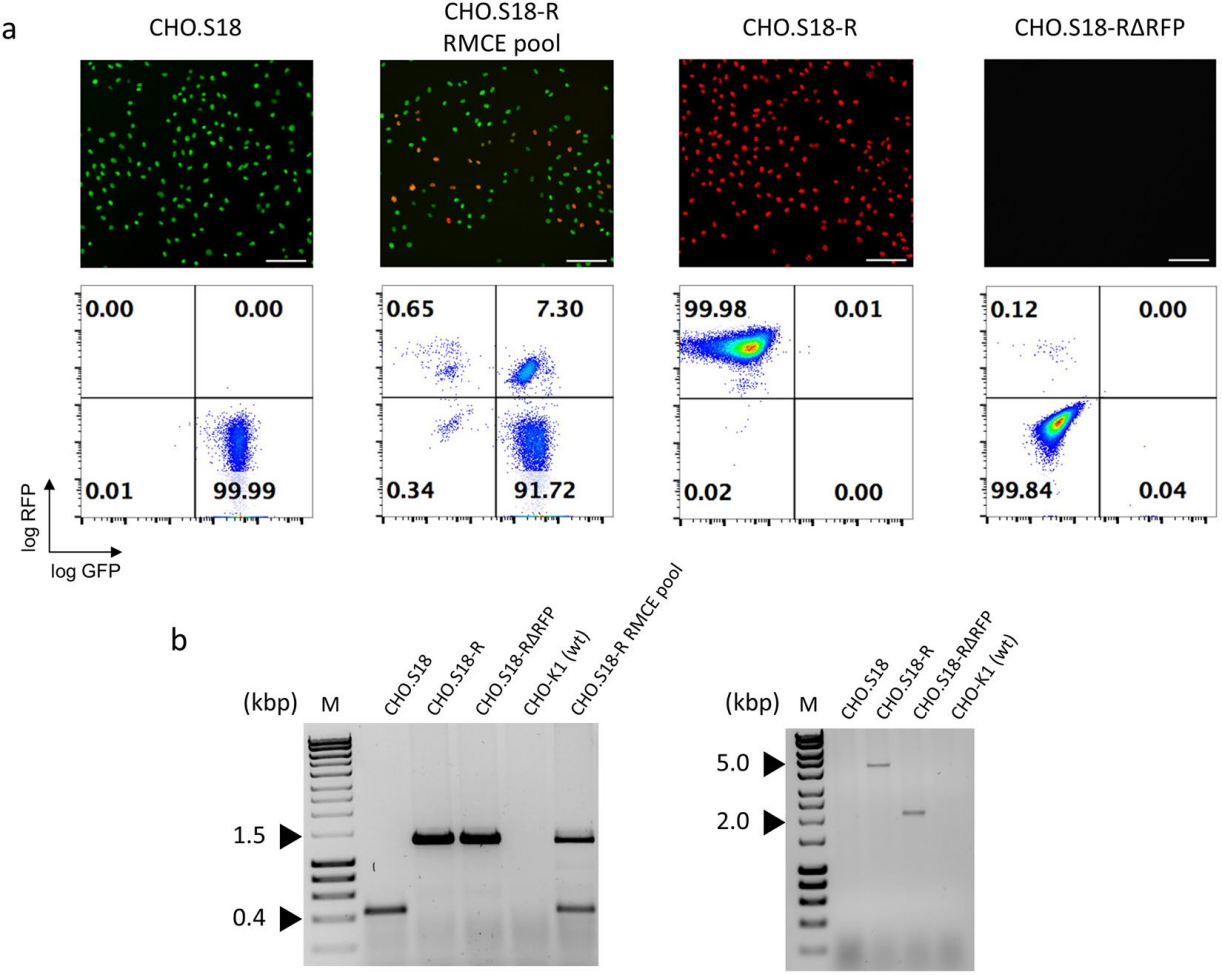
Drug selection-free RMCE. LPV-encoded GFP reporter genes in CHO cells were replaced by the DV_RMCE_ sequences. (**a**) Representative fluorescence microscopy images and flow cytometry plots of, from left to right, the CHO.S18 master clone, the unselected RMCE pool after the RMCE transfection, an isolated RFP^+^ clone (CHO.S18-R) and the same clone after Cre-mediated RFP gene deletion (CHO.S18-RΔRFP). Scale bar = 100 µm. (**b**) Verification of the expected RMCE (left) and deletion events (right) by PCR analysis of genomic DNA. The nomenclature of the cell lines and pools is according to Fig. 2a, above. Shown on the left is a multiplex PCR with 3 primers where the lower band of 537 bp is indicative of the intact LPV, whereas the upper band of 1,329 bp is indicative of the RMCE reaction. Shown on the right is a PCR analysis of integrated DV_RMCE_, before (upper band of 4,571 bp) and after (lower band of 2,157 bp) Cre/loxP deletion. For details of the PCR patterns refer to suppl. Fig. S9. (M: Hyperladder 1 kb). The uncropped gel pictures are shown in S11a.

The utility of a RMCE system for facilitating cell line engineering depends largely on the availability of high quality LPV^+^ master cell lines that allow the repeated insertion of highly expressed GOIs under standardized conditions. As the cellular background we chose Chinese Hamster Ovary (CHO) cells, one of the workhorses of recombinant protein production^15^. CHO cells were transduced with the LPV lentivirus. Clonal LPV^+^ isolates were obtained via preparative FACS/single cell deposition of GFP^+^ cells. Clones with a bright and homogenous GFP signal after initial expansion were pre-screened for a high RMCE frequency by transfection with DV_RMCE_/Flp expression vector, using the frequency of RFP^+^ cells as a first readout. A total of eleven clones that showed GFP/RFP profiles expected from RMCE occurring at either a single copy proviral LPV (two clones) or ≥ two copies proviral LPVs (nine clones) were initially identified and one clone of each of these two subgroups was characterised in more detail. GFP/RFP profiles of the DV_RMCE_/Flp expression vector-transfected CHO.S15 RMCE cell pool indicated a single LPV integration event (<0.1% recorded GFP^+^/RFP^+^ events; suppl. Fig. S2). By contrast, when CHO.S18 cells were transfected in the same way, the majority of RFP^+^ cells remained positive for GFP (Fig. 2a). Thus, more than one LPV copy must have been integrated. Subsequent analysis via next generation sequencing confirmed two independent proviral integrations in these CHO.S18 cells (see suppl. Fig. S3 for details). This clone also showed the highest phenotypic stability, as only very few GFP^−^ cells emerging after prolonged culture in absence of antibiotic selection (Fig. 2a; see suppl. Fig. S2, CHO.S15, for comparison). Aside from this stability issue, the dual LPV integration CHO.S18 line was chosen as the master clone for subsequent experiments going beyond the RMCE experiments already shown, as it allows the comparative analysis with HDR in addressing multi-loci targeting efficiency (see below). Beyond the phenotypic selection for RFP^+^ cells, we verified the accurate cassette exchange for a CHO.S18 subclone (CHO.S18-R) with integrated DV_RMCE_ by multiplex PCR specific for the expected RMCE events (Fig. 2b, left panel). The absence of the 537 bp PCR band specific for the original LPV^+^ master cell line locus demonstrated the successful cassette exchange in both chromosomal LPVs in the analysed clone, as expected from its GFP^−^/RFP^+^ phenotype. However, in a minority of the total events we examined, the GFP^−^/RFP^+^ phenotype was attributed to mixed RMCE/transgene insertion events, where in one locus the entire DV_RMCE_ was integrated via FRT_wt_. Such recombination events leave the chromosomal PuroTK gene intact and result in continued Gancicolvir sensitivity (suppl. Fig. S4). As for the GFP^+^/RFP^+^ cells with only one LPV targeted, results from a preliminary analysis by another multiplex PCR approach suggests that there is no pronounced bias for one of the two LPV copies as the preferred target for RMCE (suppl. Fig. S5). Lastly, we confirmed the possibility to eliminate the RFP transcription unit that is generated upon RMCE by Cre-mediated deletion (Fig. 1, bottom). As shown in Fig. 2b (right panel), transient expression of Cre recombinase in CHO.S18-R cells caused the expected chromosomal deletion in the resulting CHO.S18-RΔRFP cells.

### Heterologous expression of hBMP-2

**Figure 3:**
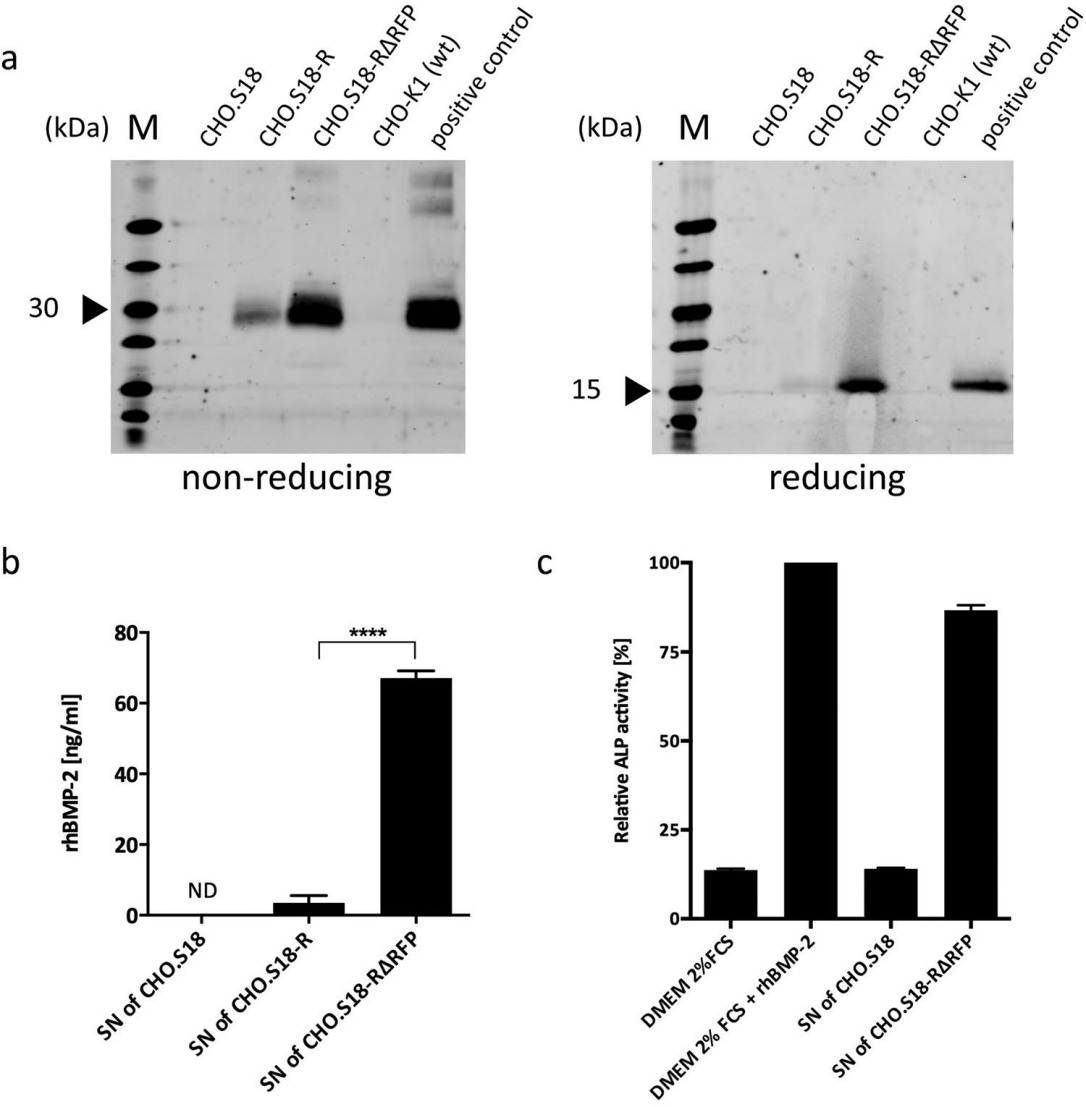
Heterologous Expression of hBMP-2. hBMP-2 was chosen as the GOI and clone CHO.S18 and its variants were characterised for hBMP-2 expression and processing. (**a**) Immunoblot analysis of supernatants (SN) of CHO.S18 clones for rhBMP-2 (M: PageRuler; Invitrogen). In SDS-PAGE under non-reducing conditions the mature dimer form of rhBMP-2 migrates at approximately 30 kDa, while under reducing conditions the rhBMP-2 migrates as a monomer of approximately 15 kDa. As positive control, CHO-K1 (wt) were transiently transfected with DV_RMCE_(hBMP-2) and medium supernatant was harvested 2 days post transfection. The uncropped blot pictures are shown in S11b. (**b**) Quantification of rhBMP-2 in supernatants of CHO S.18 clones via ELISA reveals a 19-fold increase in rhBMP-2 expression from CHO.S18-R to its RFP excision variant CHO.S18-RΔRFP. rhBMP-2 could not be detected in supernatants of parental clone CHO.S18 (ND: not detected; n = 2; Sidak’s post-hoc test was applied to detect significant differences in ANOVA, p < 0.0001; asterisks indicate a significant difference compared with the hBMP-2 concentration in the supernatant of CHO.S18-R, ****p < 0.0001). (**c**) Biological activity of produced rhBMP-2 tested by ALP assay. C2C12 cells exposed to supernatant of CHO.S18-RΔRFP containing rhBMP-2 shows an upregulation in ALP activity, suggesting a biological active form of rhBMP-2. ALP activity of C2C12 cells exposed to DMEM supplied with 2% FCS and commercial available rhBMP-2 was set to 100 (bar graphs: mean ± standard deviation; n = 3).

The GOI encoded in the particular DV_RMCE_ employed in the previous experiments, except the experiment shown in supplemental figure S1, was human Bone Morphogenic Protein-2 (hBMP-2). BMP-2 is an osteoinductive growth factor and FDA approved for the treatment of open tibial fractures and maxillofacial bones defects as well as for the induction of spinal fusions^27–29^. While we did not intend to establish a preparative experimental setting for recombinant hBMP-2 production, quantification of secretion of this growth factor was used to monitor expression of a GOI along the suggested RMCE protocol, beyond the analysis of fluorescent reporter patterns only. Immunoblot analysis for rhBMP-2 was performed for CHO.S18 cells (rhBMP-2^−^), as well as CHO.S18-R and CHO.S18-RΔRFP cells, both rhBMP-2^+^ (Fig. 3a) The secreted rhBMP-2 was apparently in its biologically active, mature dimeric form, as indicated by a blot signal that corresponded to the expected molecular weight of a dimer under non-reducing conditions. Most remarkably, when compared to CHOS18-R cells, we observed a very substantial increase of almost 20-fold in rhBMP-2 secretion for CHO.S18-RΔRFP cells, where the RFP transcription unit that preceded the hBMP-2 gene was deleted. Of note, this Cre-mediated deletion did not alter the hBMP-2 transcription unit itself (Fig. 1). This effect of strongly elevated secretion levels in CHO.S18-RΔRFP cells was also confirmed by an enzyme-linked immunosorbent assay (ELISA; Fig. 3b) for rhBMP-2 in the culture supernatant. Lastly, the biological activity of the rhBMP-2 produced by CHO.S18-RΔRFP cells was demonstrated by its ability to induce differentiation towards osteoblasts of C2C12 myoblast cells, as demonstrated by increased alkaline phosphatase (ALP) activity (Fig. 3c).

### Targeting of the LPV via CRISPR/Cas9

**Figure 4:**
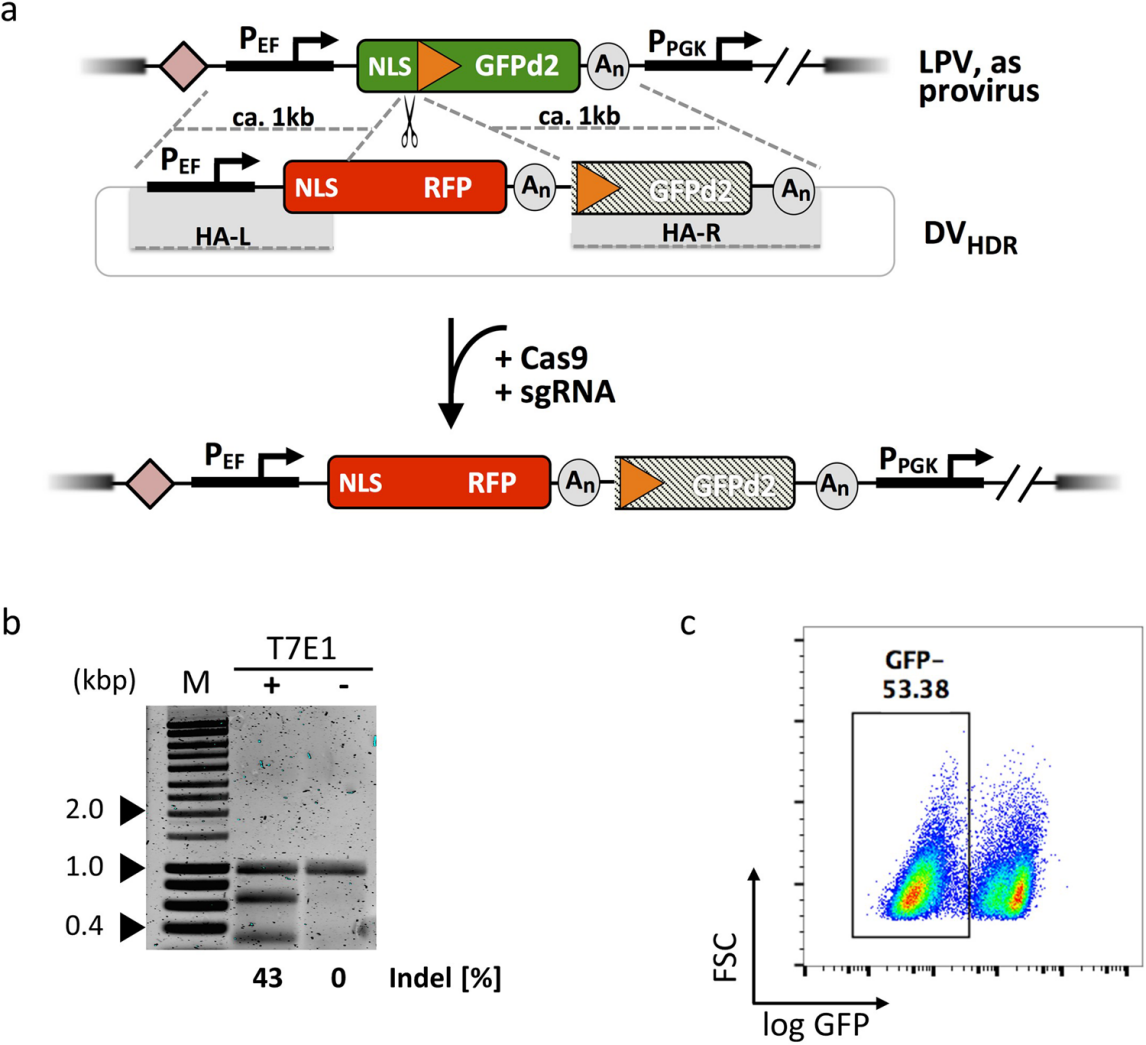
LPV-targeting via CRISPR/Cas9. Schematic overview of LPV targeting by CRISPR/Cas9 and indel efficiency test of selected sgRNA. (**a**) The chosen Cas9 cut site is located between the NLS sequence and the FRT site of the LPV. In presence of the donor vector (DV_HDR_). These double strand breaks facilitate the DV_HDR_-encoded GOI insertion via the cellular HDR pathway. The integration of the donor results in the change of the fluorescence signal from GFP to RFP. Validation of sgRNA potency: (**b**) T7E1 assay revealed efficient production of indels at the target site (M: HyperLadder 1 kb), in line with results obtained in a TIDE analysis (suppl. Fig. S10). The uncropped gel picture is shown in S11c. (**c**) Flow cytometry analysis of clone CHO.S18 after transfection with the chosen sgRNA and a Cas9 expression vector, resulting in an efficient functional knockout of the GFP reporter gene via NHEJ.

**Figure 5:**
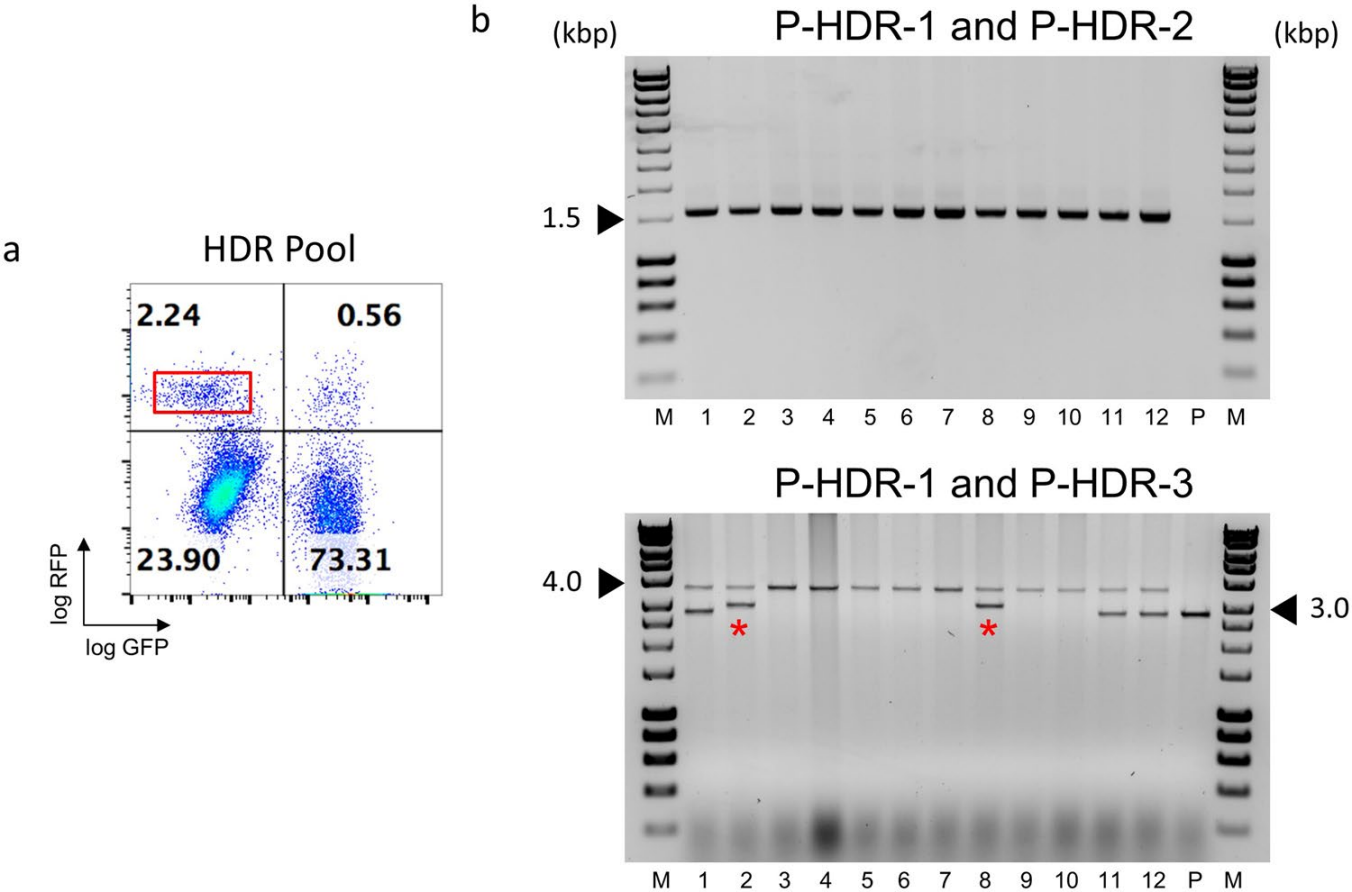
PCR characterisation of HDR events in CHO.S18. LPV sequences in RMCE master clone CHO.S18 were targeted by a co-transfection of DV_HDR_ and a Cas9/sgRNA expression vector. (**a**) Flow cytometry plot of the cell pool after transfection. The red rectangle indicates the gate used for single cell sorting for clonal isolation of GFP^−^/RFP^+^ cells. (**b**) Recombination events in 12 of the resulting clones were further characterised by PCR. In the first PCR analysis (upper panel; primers P-HDR-1 and P-HDR-2, see suppl. Fig. S9 for details) the PCR product of 1,596 bp is indicative for successful HDR events. In the second PCR analysis (lower panel; primers P-HDR-1 and P-HDR-3, see suppl. Fig. S9 for details) the entire targeted segment is amplified. Successful homologous recombination events result in an approximately 3,702 bp PCR product, whereas parental loci as well as NHEJ indels will result in a 2,825 bp PCR product. Irregular recombination events (red stars) could be detected in two cases. (P: parental clone CHO.S18). The uncropped gel pictures are shown in suppl. Fig. S11d.

We identified chromosomal loci that permit high-level, continued transgene expression in mammalian cells, which can be effectively retargeted via Flp-mediated RMCE. Such a cell line offers the opportunity to ask whether RMCE or more recently emerging technologies that allow the targeting of defined chromosomal loci with high precision have different performance characteristics. To this end, we used a CRISPR/Cas9 approach to integrate a specially designed donor vector in the LPV of CHO.S18 cells. Our experimental strategy is outlined in Fig. 4a. A sgRNA that directed efficient Cas9 cutting 3′ to the NLS of the LPV-encoded GFP was identified, monitored both by a T7E1 assay as well as the knock-out inactivation of the LPV GFP reporter gene as functional criteria (Fig. 4b,c). The position of the actual double-strand break determined the insert-proximal ends of the left and right homology arms (HA-L and HA-R, see suppl. Fig. S6 for details) in the DV_HDR_, which was tailor-made for this endonuclease site. Other than DV_RMCE_, the DV_HDR_ itself contains a functional transcription unit for the RFP reporter. This is due to the requirement for rather long homology arms, which in the case of HA-L spans over the translation start site and promoter. DV_HDR_ together with an expression vector for Cas9 and the NLS-GFP-specific sgRNA were transfected into CHO.S18 cells. After 9 days, dual parameter GFP^−^/RFP^+^ flow cytometry single cell sorting was employed (Fig. 5a). Twelve clones that gave a homogenous RFP signal upon microscopic inspection were otherwise randomly selected. Site-specific integration was analysed by PCR (Fig. 5b), as random chromosomal integration of DV_HDR_ would also result in a RFP signal. Noteworthy, we identified in all clones analysed at least one LPV targeting event. In 7/12 clones DV_HDR_ was inserted in both copies of the LPV. In 3/12 only one copy of LPV was targeted, the other apparently inactivated for GFP expression via non-homologous end joining (NHEJ). In 2/12 clones the PCR pattern indicated an irregular integration event for one of the LPV copies. Subsequent sequence analysis revealed sequence additions of CHO genomic DNA and a vector fragment of the transfected plasmid, respectively, both directly adjacent to the cutting site (suppl. Fig. S7). In summary, CRISPR/Cas9-directed HDR also allowed efficient targeting of LPV in CHO.S18 cells.

### Side-by-side comparison RMCE vs. HDR

**Figure 6:**
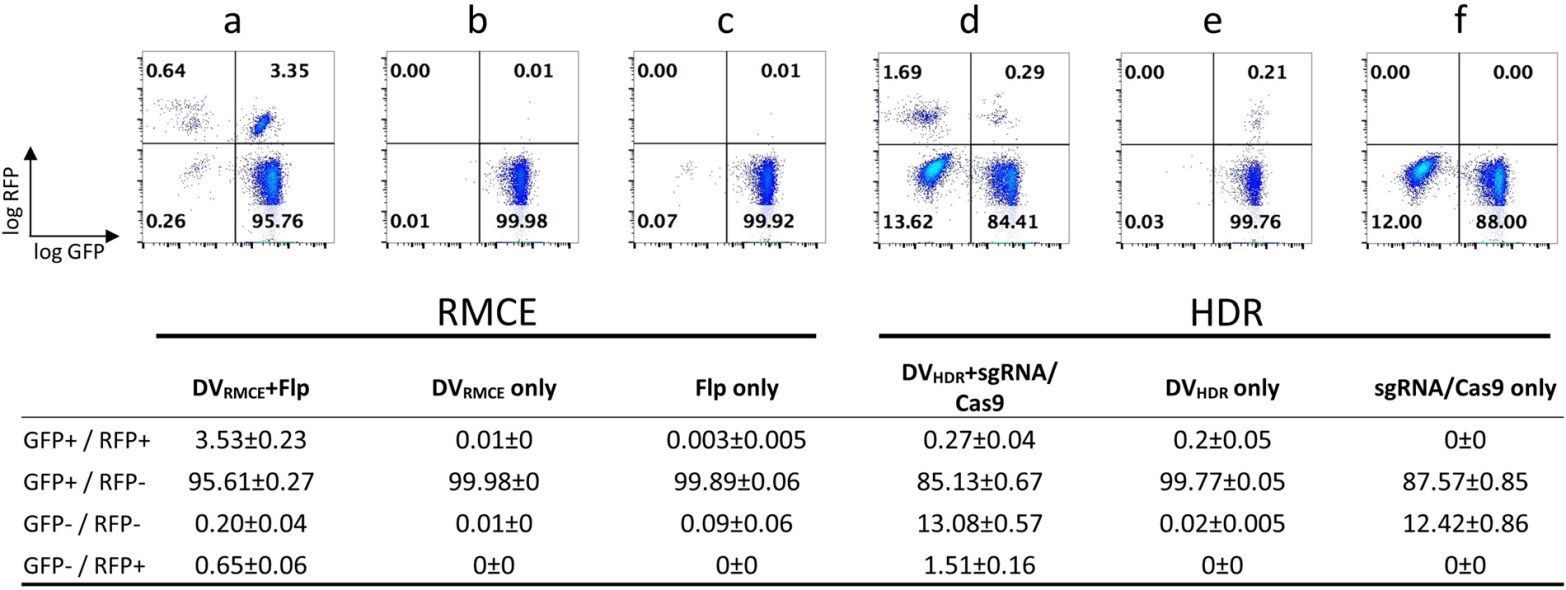
Side-by-side comparison of RMCE and HDR recombination frequencies. Both transgene integration systems targeting the same loci in cell clone CHO.S18 in a side-by-side comparison. The fluorescent marker protein distribution of unselected cell pools 9 days after transfection is shown. Below each plot the plasmids used for transient transfection are listed for both the RMCE and HDR approach. For details of the transfection reactions, see *Methods* section (summary table: mean ± standard deviation; n = 3).

The analysis of RMCE vs. HDR conducted so far pointed at principle differences in the performance of these transgene insertion systems, which were particular evident when multiple targets were present in a cell line. However, the experiments presented so far (Figs 2 and 5) were based on different transfection and cell enrichment strategies. For a more meaningful analysis of targeting efficiencies side-by-side lipofection of the CHO.S18 LPV^+^ line was performed. While keeping the plasmid inputs comparable, no subsequent antibiotic selection or enrichment of transfected cells by cell sorting was employed, and cells were grown out for 9 days. As shown in Fig. 6, transfection of CHO.S18 cells with only DV_RMCE_ did not yield, as expected, any RFP^+^ cells (Fig. 6b), as DV_RMCE_ requires Flp-mediated LPV targeting for activity. In contrast, transfection of the intact transcription unit contained in DV_HDR_ resulted in about 0.2% RFP^+^ cells (Fig. 6e). Given a transient transfection efficiency of >30% in this series of experiments we observed a stable, most likely random transgene integration of less than 1% of the successfully transfected cells. Expression of Flp alone in the CHO.S18 cells resulted in a low but distinct (<0.2%) population of GFP^−^ cells (Fig. 6c), which might reflect residual recombination between FRT_wt_ and FRT_F3_ sites^12^. As expected, in absence of DV_HDR_ co-expression of Cas9 and the NLS-GFP-specific sgRNA resulted in a functional knockout via NHEJ of the LPV target, here in about one third to half of the actually transfected cells (Fig. 6f). Transfection in a RMCE setting (DV_RMCE_ + Flp) resulted in targeting events (i. e. RFP^+^ cells) in 4 to 5% of the total cells (Fig. 6a). However, only in about one fifth of those cells both GFP transgenes were inactivated (GFP^−^/RFP^+^). In sharp contrast, whereas transfection in a HDR setting (DV_HDR_ + sgRNA/Cas9) resulted in somewhat less efficient LPV targeting (in 1.5 to 2% of the total cells), in >80% of those recorded events both of the LPV loci were hit, either by insertion or knock-out (Fig. 6d). However, while these results provide a rough estimate of overall engineering efficacies, we also like to stress the limitations of this semi-quantitative comparative analysis. Not only might results substantially differ when using other cell lines, but also the exact ratio of recombination donors and expression levels for recombinases/nucleases plus sgRNAs have to be taken into account. Nevertheless, the focus on identical target sites for transgene integration in the exemplary CHO.S18 master cell line, and largely similar results for other multi-LPV clones analysed in the course of this study, provides valuable insights in performance characteristics of both the genome engineering systems analysed.

## Discussion

Progress in genome engineering technology results in a constantly increasing choice of available tools, which often lack direct comparison, both quantitatively and qualitatively. Given the different underlying modes of action of the enzymes employed, one would not necessarily expect one of these tools to emerge as the “one-serves-all” solution. Here we explored a Flp recombinase-based cassette exchange mechanism and Cas9-based, HDR-mediated transgene insertion in an identical recipient cell line, in which the same insertion loci were targeted. Initially established for a RMCE protocol, our experimental design allowed for the rapid and efficient isolation of targeting event by following a switch in fluorescent protein expression. The strategy pursued by us works efficiently even without the use of any supportive antibiotic selection. Cell lines created by drug selection protocols often show heterogeneous transgene expression patterns and silencing of the transgene over time^18^. Especially in combination with a growth disadvantage for high expressing cells, this can lead to a decrease in transgene expression levels^30,31^. Due to dynamic epigenetic effects that manifest themselves also in dependence of cell types, the nature of transcription signals, integration sites and other variables, these problems often persist even after isolation of cell clones with initially favourable characteristics^32,33^. In this current study the donor vector contained no antibiotic selection marker. While the design of the landing pad vector allowed for an antibiotic-based positive/negative selection scheme^12,23,34^, such a selection was not an integral part of our routine cell line engineering protocol. Instead, desired features of transgene expression like stability of transgene expression over time and homogeneity served as integral parts of the clonal isolation strategy. While we did not systematically analyse RMCE cell pools for genetic homogeneity and phenotypic stability, both the PCR and analytical flow cytometry results suggested that at least for some applications omission of clonal isolation would not compromise transgene expression in a major way. Other antibiotic-free protocols for cell line engineering rely on the recombinase-mediated deletion of fluorescence markers directly linked to transgene activation. Following this paradigm we previously generated clones and showed uniform expression patterns as well as remarkably high-level of transgene expression over a time course of 100 population doublings^18^. Alternatively, techniques like the cold-capture selection method employ the transiently enhanced accumulation of the secreted protein of interest itself on the cell surface for isolating high-producing cell lines with good success^35,36^.

Subsequent to the analysis of the DV_RMCE_ integration in the CHO cells we quantified the expression of hBMP-2 gene, chosen as the GOI for this study. Most strikingly, we found a very substantial increase in GOI expression (almost 20-fold) after the loxP/Cre-mediated excision of the RFP transcription unit. We consider implausible that under the given experimental conditions and without an observed change in growth parameters the relief from an extra consumption of metabolic resources (also referred to as “metabolic burden”^37,38^) caused the increase in hBMP-2 synthesis. Transcriptional interference in the tandem constellation of promoters before RFP deletion is likely to contribute to the observed effect^39^, despite the separation of the transcription units by an effective termination signal^40^. It remains unclear, though, what other factors might influence the steep increase in GOI expression. However, the possibility to insert modified DVs to the landing pad provides the opportunity for a mechanistic analysis of the observed effects and has to be addressed in future experiments. Using the same transgene expression and RMCE approach in an unrelated research project, we observed that the expression of hOct4 in an macrophage precursor cell line also increased to a comparable extent when the 5′ preceding fluorescence marker gene was deleted (suppl. Fig. S8). Thus, while the magnitude of the RFP deletion-effect on hBMP-2 expression might be related to our vector design, it is neither cell type nor integration site specific.

Next we asked whether using Cas9 to facilitate HDR-mediated transgene insertion in a landing pad is able to compete in terms of efficacy with Flp in a LPV^+^ cell line optimized for RMCE. Obviously, RGEN technology can satisfy experimental demands that cannot be addressed by recombinases, like direct gene knock-outs or gene repair. Also with respect to transgene insertion/replacement RGENs and other programmable nucleases could be advantageous for directed chromosomal insertions, as for several applications this strategy might allow for cell line engineering without prior establishment of master cell lines. The targeting of the LPV loci by a CRISPR/Cas9 approach required the design of a different donor vector (DV_HDR_) with suitable homology arms. As with DV_RMCE_, use of this vector allowed to monitor successful integration events by loss of the GFP signal and a concomitant gain in RFP signal. However, a random integration of DV_HDR_ would also result in a RFP signal. In practice though, this turned out not be a major limitation. Under unselected experimental conditions the Cas9-facilitated HDR process was about tenfold more frequent than random integration (see Fig. 6, HDR panels: DV_HDR_^+^ sgRNA/Cas9 vs. DV_HDR_ only). The chosen experimental setting allowed for a direct comparison, Flp *vs*. Cas9, of integration frequencies at the LPV loci. According to the isolation of the LPV^+^ master cell line we expected the fluorescent protein signals from integration sites to favour not only high and stable transgene expression but also high recombination efficiency. This interrelation has been described in more detail before^19,41^. However, it is understood that any absolute comparison in integration efficiency beyond our use of identical target sites would require a systematic experimental optimisation. One would have to address the available nuclear amounts and ratios of donor vector and recombinase (in case of RMCE) or sgRNA-loaded Cas9 protein (in case of HDR). Previous work demonstrated the importance of this aspect for both HDR^42,43^ and RMCE^44,45^.

Contrary to the HDR reaction, Flp-mediated recombination does not require additional host factors, nor does it involve nucleolytic activities or DNA synthesis. Thus, one might consider recombinase-mediated DNA insertion a “mild” form of genotoxic stress, whereas both NHEJ and nuclease-facilitated HDR involve the generation of DNA double-strand breaks with free DNA ends^2^. This processing is instrumental for knockout strategies using RGENs on the one hand, but might be an issue for HDR-mediated genome engineering. Notably, aberant recombination was observed in 2 out of 12 cell clones categorized by us as successful recombinants according to phenotypic criteria. Both cases involved the integration of additional DNA sequences in the immediate vicinity of the sgRNA/Cas9 cut site. Of note, in one case the additional sequence originated from an input plasmid, while in the other affected clone the extra DNA was a chromosomal hamster DNA fragment. Such aberrant events were observed before for CRISPR/Cas-mediated gene insertions^46^. They may have little practical implications for biotechnologically driven cell line engineering and its relative ease of validation, but are worth further attention in more complex experimental settings, including gene repair strategies.

The most striking result of the comparative part of the analysis presented here was the differences between Flp- and Cas9-mediated genome engineering in targeting multiple copies of a given transgene, here the LPV present in two copies in the master cell line. For Cas9-facilitated HDR, the majority of the *bona fide* integration events result in the targeting of both loci. Note that the RFP^+^/GFP^−^ phenotype was not necessarily indicative of DV_HDR_ insertion in both transgenes (PCR analysis Fig. 5: 7/12), but can be a mixture transgene insertion in one copy and knock-out of the other LPV copy via NHEJ (PCR analysis Fig. 5: 3/12). This is in sharp contrast to our results for RMCE (or in rare cases DV_RMCE_ insertion), where the simultaneous targeting of both loci is rather the exception. This principle difference between the two targeting technologies extended to all CHO.S-type clones with more than one LPV that were analysed by us. A high efficiency to simultaneously engineer both alleles of a given gene in mice via Cas9 has been described, both for gene knock-outs^47^ as well as HDR-modifications^48^ and is mirrored by the high efficiency in multiplexing strategies that involve the simultaneous use of multiple sgRNAs at the same time^49,50^.

Notwithstanding the rising popularity or RGEN technology, targeted transgene insertion by site-specific recombinases like Flp remains a powerful genome engineering tool. Recent efforts in redirecting recombinases to endogenous chromosomal sites, either via protein evolution approaches^51,52^ or fusion to designer DNA binding moieties^53^ might develop into attractive alternatives for ever more efficient and reliable genome engineering in biotechnology and biomedicine.

## Methods

### Gene synthesis, cloning procedures and vector nomenclature

Synthetic DNA fragments used for the generation of the LPV and the different DVs were provided by Entelechon GmbH and assembled using standard cloning procedures. All restriction enzymes and cloning kits were purchased from New England Biolabs (NEB) and used accordingly to the manufacture’s instruction. As for the nomenclature, only one LPV construct and one DV_HDR_ construct were employed in the study and are referred to accordingly. By contrast, three different DVs were employed in the analysis of RMCE, with DV_RMCE_ used as a generic name, and DV_RMCE_(MCS), DV_RMCE_(hBMP-2) and DV_RMCE_(hOct4) to specify the detailed GOI/MCS where appropriate.

### Cell culture

CHO-K1 (ATCC:CCL-61) and CHO-derived stable cell lines were cultivated in RMPI 1640 medium (Gibco) supplemented with 10% heat inactivated FCS and 100 µg/ml of penicillin/streptomycin in a humidified incubator at 37 °C with a 5% CO_2_ atmosphere. HAFTL cells^54^ were cultured under the same conditions, but medium was further supplied with 50 mM beta-mercaptoethanol (βME). C2C12 cells (ATCC: CRL-1772) were cultivated in DMEM supplemented with 10% FCS, 2 mM Glutamine, 100 µg/ml of penicillin/streptomycin. HEK 293TN (System Biosciences; LV900A-1) were cultivated in Advanced DMEM (Gibco) supplemented with 2% FCS, 0.01 mM cholesterol, 0.01 mM lecithin and 1:100 chemical defined lipid concentrate. Fluorescence microscopy image acquisition was done by the Axio Observer.Z1 (Zeiss) and its imaging software AxioVision 4.7.2 (12-2008).

### Analytical flow cytometry and cell sorting

Cells for analytical flow cytometry and FACS sort were prepared as previously described^55^. Flow cytometry analysis was done on the Becton Dickinson (BD) Accuri C6 flow cytometer (Filter: GFP 533/30 and RFP 610/20) and for preparative FACS, cells were sorted with BD FACSAria II.

### Lentiviral packaging and titration

Lentiviral vectors were packaged in HEK293TN cells and virus titer was determined as previous described^56^. Cell culture supernatant containing recombinant virions were filtered through 0.45 µm filter and concentrated using Lenti-X (Clontech) following the manufacturer’s instruction.

### LPV cell line creation

LPV cell lines were generated by lentiviral transduction. The MOI was adjusted to 0.1 to ensure low number of integrated proviruses. Cells were seeded in a 12-well plate with growth medium supplemented with 8 µg/ml polybrene (Sigma). 24 hours after seeding, lentiviral particles were added to the cells and incubated for 16–18 hours before medium change. GFP positive cells were sorted using FACSAria single cell deposition^18^. Clones were picked and analysed via flow cytometry and selected based on their strength, homogeneity and persistence of the GFP signal.

### PCR and T7E1 assay

PCRs for cloning and T7 Endonuclease I (T7E1; NEB) assay were performed with the Q5 PCR Mastermix kit. For PCRs with amplicons longer than 3 kbp, the LongAmp Taq PCR kit was used. Both PCR kits are distributed by NEB and optimal annealing temperatures were determined by the NEB Tm Calculator (http://tmcalculator.neb.com). PCR programs were set according to the manufacturer’s instructions. T7E1 assays were performed as described^57^. Cleaved DNA fragments were separated on a 2% agarose gel and stained with Syto60 staining solution (1:9,000) for 45 minutes while shaking. Gels were analysed on the Odyssey infrared imaging system (Li-cor) using the manufacturer’s software and the DNA concentration of each band was quantified using the ImageJ software. Percent values of indels were calculated as described^49^.

### Transfections

Prior to stable RMCE experiments, the RMCE strategy was tested in transient transfections. HEK293TN cells were co-transfected with DV_RMCE_, LPV, and a Flippase plasmid. This Flp expression vector was derived from of pCAGGS-Flpe-puro (Addgene #20733) and modified by substituting the puromycin resistance gene by a blue fluorescent protein (BFP) marker, facilitating the isolation of transfected cells by preparative FACS, if desired. One day prior to transfection, 4 × 10^5^ HEK293TN cells per well were seeded in a 6-well plate. A total of 2 µg plasmid DNA was mixed in a mass ratio of 1:1:1 in 150 mM NaCl and combined with a 7,5 mM polyethylenimine (PEI) solution, at a nitrogen/phosphate (N/P) molar ratio of 10. The mix was incubated for 10 minutes at room temperature and added dropwise to the cells.

For stable RMCE experiments and sgRNA/Cas9 efficacy tests, CHO-K1 and HAFTL target cell lines were transfected by electroporation. Cells were resuspended in 600 µl of antibiotic-free medium and electroporated in 0.4 cm cuvettes with the Gene Pulser Xcell from Biorad (CHO cells with square wave pulse protocol: 250 V, 15 ms, 2 pulse, 0.1 s pulse interval; HAFTL cells with exponential decay wave pulse protocol: 300 V, 950 µF capacitance). 20 µg of DV_RMCE_ with 20 µg of Flippase vector were used. In this experiment, transfected BFP^+^ cells were isolated using preparative FACS. RFP deletion was mediated by Cre recombinase. Here, 40 µg of pPGK-Cre-bpA (gift from Klaus Rajewsky (Addgene plasmid #11543)) was electroporated into the selected CHO and HAFTL cell clone with the indicated pulse conditions. Transfected cells were kept in subculture for 2 weeks and a RFP^−^ population was isolated via FACS.

For sgRNA efficacy test 20 µg of pX330-sgRNA:NLS-GFP (sgRNA/Cas9 vector; derived from Addgene #42230) with 20 µg of salmon sperm DNA were used for each transfection. For HDR transfections as well as the side-by-side comparison with RMCE, CHO cells were transfected with Lipofectamine 3000 (Invitrogen). In a 6-well plate, 1.5 × 10^5^ cells were seeded a day prior to transfection. Either 1 µg of sgRNA/Cas9 or Flippase plasmid and the 1 µg of the appropriate DV were mixed and transfected accordingly to the manufacture’s instructions.

### Next generation sequencing

For the library preparation, 2.5 µg genomic DNA of CHO.S18 was digested overnight with the restriction enzymes BamHI, BglII, EcoRI, MfeI, and HindIII in a total volume of 100 µl. The digest was purified via NucleoSpin Gel and PCR Clean-up (MACHEREY-NAGEL) and linkers were ligated to the DNA ends overnight with T4 DNA Ligase. After heat inactivation at 70 °C for 15 minutes, 1 µl of each ligation was used as the first round PCR template for amplification with one primer complementary to the linker DNA and one complementary to the 3’end LTR of the HIV provirus. The secondary PCR added the indices needed for sequencing on the MiSeq sequencer (Illumina). Methods are essentially as described (Genome Walker Kit, Clontech) using primers described in the supplements. For CHO.S18, HIV-1 LTR specific reads covering the proviral integration boundary for 2 locations were obtained.

### Immunoblot analyses

#### Sample preparation for rhBMP-2 detection

For immunoblot analysis of rhBMP-2, supernatants were filtered (0.45 µm) and proteins were precipitated by adding 1 volume of 100% (w/v) trichloroacetic acid (TCA) to 10 volumes of filtered supernatant, incubation on ice for 1 hour and centrifugation at 18,000 × g for 15 min at 4 °C. Supernatants were discharged, the protein pellets washed in 1 ml ice cold acetone and centrifuged at 18,000 × g for 15 min at 4 °C. Air-dried pellets were resuspended in RIPA buffer (50 mM HEPES-KOH, 500 mM LiCl, 1 mM EDTA, 1% NP-40, 0.7% sodium deoxycholat) by sonication with a BANDELIN SONOPULS HD 2070 at an amplitude setting of 90% for 2 minutes.

#### Sample preparation for hOCT-4 detection

HAFTL cells were centrifuged at 300 × g for 5 minutes at room temperature, cell pellets resuspended in RIPA buffer and incubated on ice for 15 minutes. Lysed cells were sonicated as described above and subsequently centrifuged at 15,000 × g for 15 minutes at 4 °C.

#### Immunoblotting

Protein samples were diluted in RIPA buffer, mixed with loading buffer (10% glycerol, 50 mM Tris pH 6, 8, 2 mM EDTA, 2% SDS, 0.02% bromophenol blue, 5% βME (omitted when samples ran under non-reducing conditions)). PAGE separation, protein transfer and detection were done as previously described^55^. For detection of rhBMP-2 and hOCT-4, a rabbit anti-BMP-2 antibody (Dianova CYT-26591; 1:5000) and a goat anti-OCT-4 antibody (Santa Cruz sc-8628; 1:2000) were used. Total proteins blotted onto nitrocellulose membranes were stained by colloidal gold^58^.

### hBMP-2 ELISA

rhBMP-2 concentration in medium supernatants of CHO.S18 clones and its variants were quantified by ELISA according to the instruction of the manufacturer (Antigenix America Inc.; RHF913CKC). Absorption was measured with a SpectraMax 340PC384 at a wavelength of 405 nm. A standard curve was derived using recombinant hBMP-2 was and the concentration of expressed protein in the samples was determined by linear regression.

### Biological activity of rhBMP-2 and ALP assay

hBMP-2 is able to suppress myoblast differentiation in the myogenic cell line C2C12. At the same time it promotes osteoblastic differentiation, which results in the expression of alkaline phosphatase (ALP)^59^.

C2C12 cells were exposed to the medium supernatant of clone CHO.S18-RΔRFP containing rhBMP-2 and ALP activity was determined. On a 96-well plate, 10.000 C2C12 cells were seeded per well and differentiation protocol was started 24 hours after seeding. Growth medium was exchanged for depletion medium (DMEM with 2% FCS, 100 µg/ml of penicillin/streptomycin, and 2 mM L-glutamine). Further, medium was mixed with supernatant from CHO.S18-RΔRFP to reach the end concentration of 1 nM rhBMP-2. Human BMP-2 (InductOS) served as the positive control and was added in the same concentration. As the negative control, supernatant from parental clone CHO.S18 was added in the same volume as for CHO.S18-RΔRFP. Medium was changed every other day over the time course of 8 days. Subsequently, differentiated C2C12 cells were tested for ALP activity via a colorimetric assay based on the dephosphorylation of para-Nitrophenylphosphate (pNPP) as described previously^60^. Absorption was measured with the SpectraMax 340PC384 at a wavelength of 405 nm.

### Statistical analyses

Data are represented as mean ± standard deviation. Unpaired t-test as well as One-way ANOVA followed by Sidak’s multiple comparisons test were performed using GraphPad Prism version 6.00 for Mac, GraphPad Software, La Jolla California USA, www.graphpad.com.

### Data availability

The datasets generated during and/or analysed during the current study are available from the corresponding author on reasonable request.

## Electronic supplementary material

Supplemental Information for ″Site specific chromosomal gene insertion: Flp recombinase versus Cas9 nuclease″
