# Supplementary material for: Site-specific chromosomal gene insertion: Flp recombinase versus Cas9 nuclease
Source: Sci Rep. 2017 Dec 19;7:17771. doi: 10.1038/s41598-017-17651-0 (PMC5736728; doi:10.1038/s41598-017-17651-0)
Supplement: Supplementary file 1 — Supplemental Information for ″Site specific chromosomal gene insertion: Flp recombinase versus Cas9 nuclease″ [file 41598_2017_17651_MOESM1_ESM.pdf]

**Supplemental Figures (S1 – S11) and Supplemental Table 1**

# Site-specific chromosomal gene insertion: Flp recombinase *versus* Cas9 nuclease

Quang Vinh Phan, Jörg Contzen, Petra Seemann & Manfred Gossen

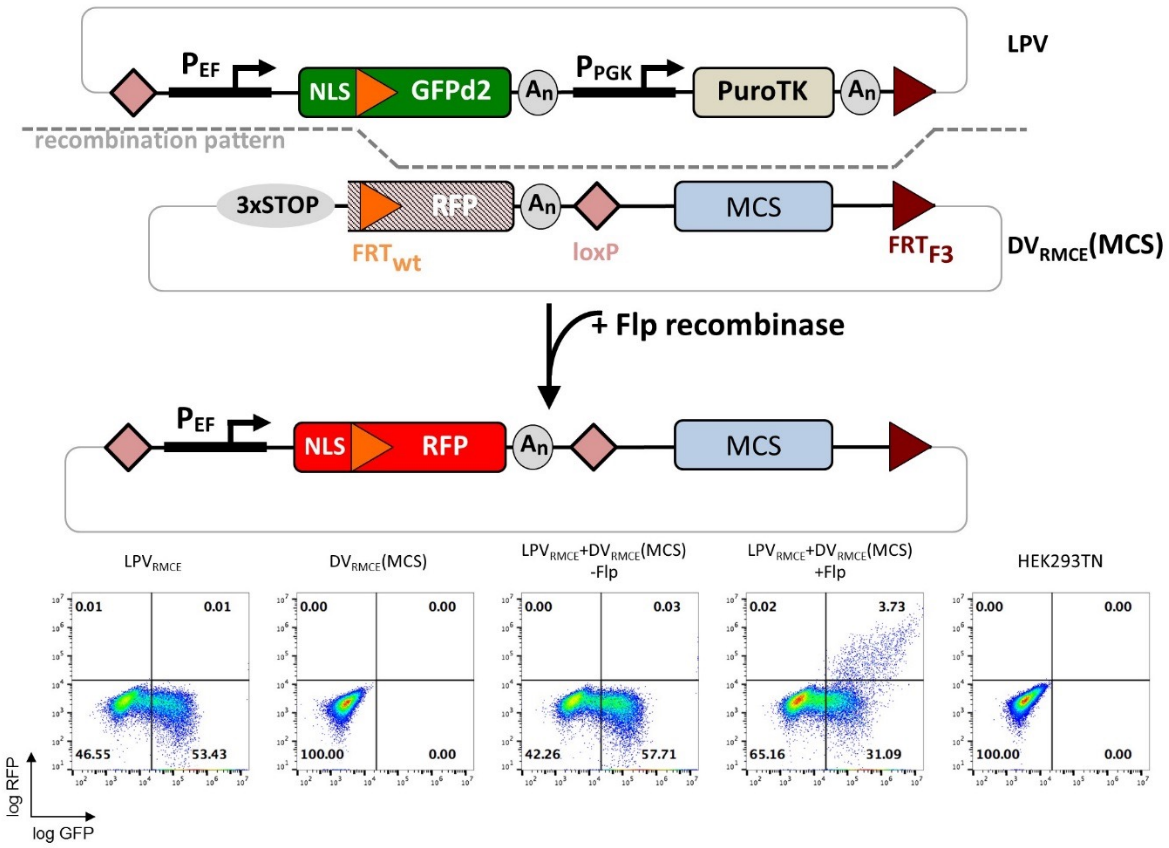


**Supplemental Figure S1: RMCE in transient transfections**

HEK293TN cells were transfected with $LPV_{RMCE}$, $DV_{RMCE}$(MCS), and pCAGGS-Flpe-puro (scheme upper panel). Thus, recombination takes place on a plasmid level and successful recombination events are indicated by the occurrence of a RFP signal. As expected, only the combination of all three plasmids together yielded in a RFP positive population. The flow cytometric analysis shown in the lower panels was performed 2 days after transfection.

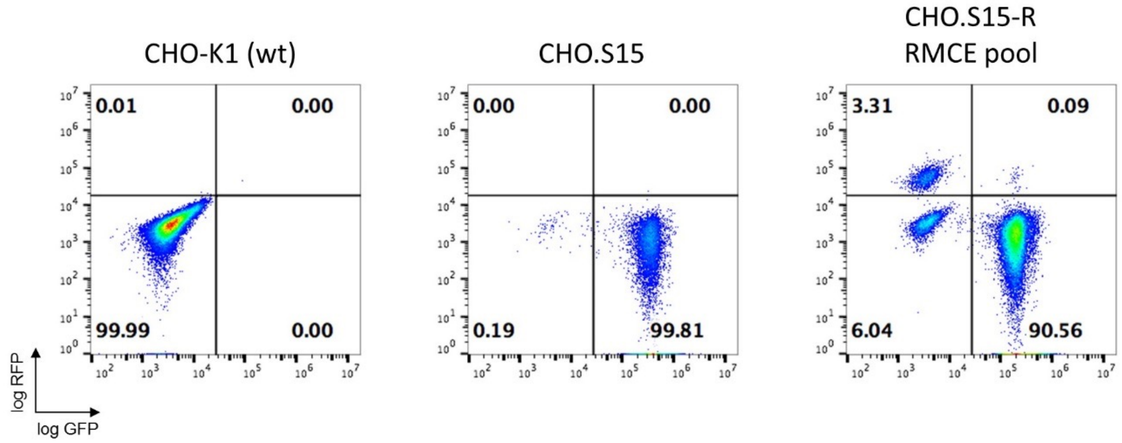


**Supplemental Figure S2: Flow cytometry analysis of CHO.S15**

Flow cytometry profile for GFP and RFP of CHO.S15 cells before and after RMCE. The fluorescent protein expression pattern of the RMCE cell pool is indicative for a single copy of the LPV as a single RMCE reaction resulted in a distinct $RFP^+/GFP^-$ population and almost no double positive cells.

BLAST result:
Cricetulus griseus strain 17A/GY chromosome 1 unlocalized genomic scaffold chr1_scaffold_250, whole genome shotgun sequence

ATGACCAGGAGTAGGGATTTGTGAGCTCTCCCATCTATGTCTGTTCTCATTGTATAGCTCTTGTTTAGGAGAGCACGTTTTAGGCTTTCATGGGTGCAGCCTAGGTGTCATAATTAGAGCACA
CTGTCTCACAGCAGCCAGCCTGCTCCTGTGGCTTTTACAGTCTTTCCACCCCGTCTTCCGCAACGCCCCCTGAGCCGCAGATTGTGTTGTAGACATATCAGTTGGGTTTCCACACTCAGTTCTT
GACTTTTGGGCCAGGAGTAGGTTCCTGTGACAGTCTCCATTTTCTTTTTTCTTTAATCCTTTCTGTTCAACGAAACCTTCTTTAAAGGCCCCCTCCTCCAGAAGACCTCATGACTTCCACCCTGG
AAGGGCTAATTCACTCCCAACGAAGACAAGATATCCTTGATCTGTGGATCTACCACACACAAGGCTACTTCCCTGATTGGCAGAACTACACACCAGGGCCAGGGATCAGATATCCACTGACC
TTTGGATGGTGCTACAAGCTAGTACCAGTTGAGCAAGAGAAGGTAGAAGAAGCCAATGAAGGAGAGAACACCCGCTTGTTACACCCTGTGAGCCTGCATGGGATGGATGACCCGGAGAG
AGAAGTATTAGAGTGGAGGTTTGACAGCCGCCTAGCATTTCATCACATGGCCCGAGAGCTGCATCCGGACTGTACTGGGTCTCTCTGGTTAGACCAGATCTGAGCCTGGGAGCTCTCTGGCT
AACTAGGGAACCCACTGCTTAAGCCTCAATAAAGCTTGCCTTGAGTGCTTCAAGTATGGGGGG...

5'end

-LPV Provirus-

...AGCCGCCTAGCATTTCATCACATGGCCCGAGAGCTGCATCCGGCCTGTACTGGGTCTCTCTGGTTAGACCAGATCTGAGCCTGGGAGCTCTCTGGCTAACTAGGGAACCCACTGCTTAAGC
CTCAATAAAGCTTGCCTTGAGTGCTTCAAGTAGTGTGTGCCCGTCTGTTGTGTGACTCTGGTAACTAGAGATCCCTCAGACCCTTTTAGTCAGTGTGGAAAATCTCTAGCACACCCTTTCCCAT
CTTTTAATGTTTCTGACTATACTTACATTATTTTTGTTTGTTTTGTTATTTTTTGAGACAGGTCTCGTGTAGCCCAGGTTGGCCTTGAACTCATTATATAGCCAAGGATAACTTTGAGCTCCTGAT
CCACCTATCTCTGCCTCTCAAGTGTTGTCATTACATGTATGCAACATTGCACTGGGCTCTGCACCCTATTTAAGGGCACAAACATCCCACCAAGTGTTACTGGATCATCTGCAGTACTACTGTG
CTTTTGCTTGTGTAGTGTTCTGCTCCTGTGTGTTGTCTGACAGTGAGCCATGTGCAGCCCG

3'end

BLAST result:
Cricetulus griseus strain 17A/GY chromosome 4 unlocalized genomic scaffold chr4_scaffold_68, whole genome shotgun sequence

ATATTAAGAAGTAAAATTTCAAATATGGACACTTAGCATATAACATTTTCACTTTCCCCCACCCTTTTTTGTGAGACCAAGTCTTATTACATAAATCAAGCTGACTTGGGACTCATGGTGATCA
CCCTGCCTCAGCTCCCAGACATTTTAAAAATAGAAGTTCCCATCTACATCTTCCCTGCAAGGCTCAGGGAAGTGTAACAGAGGAGAGCAGAGGTTGTAAGAGCTGGAGGAACCACAATGCG
GACACTGTCCTCTAGGTATGAGTTGCCCACAGCCTTGTGACCCAGAGCAGCTGTGATTACCCACATAAGACTTGTACAAGGTTGGGCTGTTAGCATTCCTTCATGGGACCAGGGAGGGGCTC
AGGAGGCCTGGCCCATCCCTGAGTGTTTATTTATAGGTGGTTAATGTTTTCTGGGGAAGCAGAAGATGCTTTCTTCAGTCATATAACCACTTGTAAGTGGAAGGGCTAATTCACTCCCAACG
AAGACAAGATATCCTTGATCTGTGGATCTACCACACACAAGGCTACTTCCCTGATTGGCAGAACTACACACCAGGGCCAGGGATCAGATATCCACTGACCTTTGGATGGTGCTACAAGCTAG
TACCAGTTGAGCAAGAGAAGGTAGAAGAAGCCAATGAAGGAGAGAACACCCGCTTGTTACACCCTGTGAGCCTGCATGGGATGGATGACCCGGAGAGAGAAGTATTAGAGTGGAGGTTT
GACAGCCGCCTAGCATTTCATCACATGGCCCGAGAGCTGCATCCGGACTGTACTGGGTCTCTCTGGTTAGACCAGATCTGAGCCTGGGAGCTCTCTGGCTAACTAGGGAACCCACTGCTTAA
GCCTCAATAAAGCTTGCCTTGAGTGCTTCAAGTATGGGKKGCCCGTCA...

5'end

-LPV Provirus-

...AGCCGCCTAGCATTTCATCACATGGCCCGAGAGCTGCATCCGGACTGTACTGGGTCTCTCTGGTTAGACCAGATCTGTGCCTGGGAGCTCTCTGGCTAACTAGGGAACCCACTGCTTAAGC
CTCAATCAAGCTTGCCTTGAGTGCTTCAAGTAGTGTGTGCCCGTCTGTTGTGTGACTCTGGTAACTAGAGATCCCTCAGACCCTTTTCGTCAGTGTGGAAAATCTCTAGCAGTAAGTTGGTCA
TGTTCCTATAAACAACTATATACCAATGTTCTTGTAGTAACCCTATTAAACCCACAGCGTCATAAAGGAAAGAGAGAAAGGAAAGAAAGAAAGGACAGACAGATCTAAGGAAAGATCCTGA
TCATATTAAAAAATAAAATTAGTGGCTTTCATTTTTCTAATCCTTAAATGAAAAAGACGTAAAGGACGATACAGATTAAAAGACCGGAAAGATTCAGTAAGCCAAACTCTTCTTTGAACTGA
GTGGCAGCAGGGAGAACGCGCAGCCCG

3'end

**Supplemental Figure S3: LPV integration sites in clone CHO.S18**

Sequencing data for the 5' and 3' end cover the proviral integration boundaries (proviral sequences marked as red). BLAST search (BLAST assembly build: GCA_000448345.1) of these two sequences revealed integration events in chromosome 1 and 4.

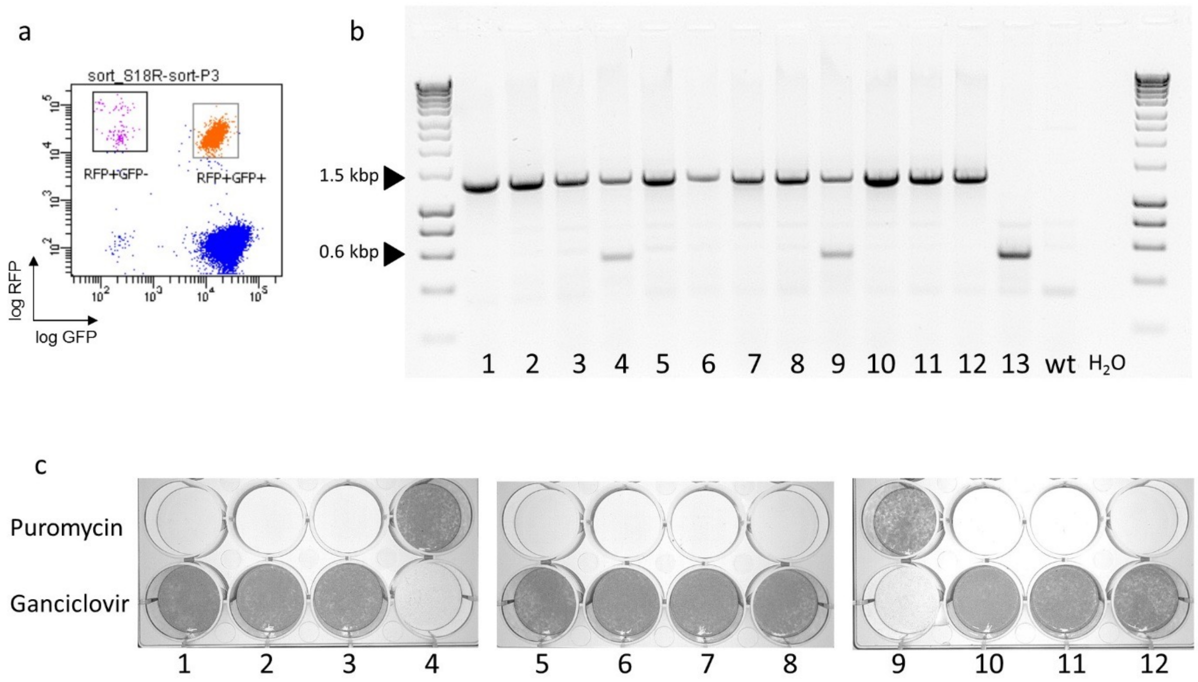


**Supplemental Figure S4: RMCE and Flp-in events within the CHO.S18-R RMCE pool**

The co-transfection of the RMCE donor and Flp plasmid can also facilitate a Flp-in event, where the RFP marker with the whole vector backbone is integrated via the $FRT_{wt}$ site without the removal of the LPV cassette containing the GFP and the PuroTK selection marker. (a) To assess the extent of this particular recombination event, we single cell-sorted from the CHO.S18-R RMCE pool for $RFP^{+}/GFP^{-}$ cells. (b) Integration patterns of 12 randomly selected clones were analysed via PCR. The same primer set and PCR conditions were used as in Figure 2b. The uncropped gel picture is shown in suppl. Fig. S11e. The amplicon at 537 bp indicates the presence of the PuroTK cassette in clone 4 and 9. Labeling of the lanes: 12 clones (1-12), parental clone CHO.S18 (13), CHO wt cells (wt) and addition of water only as the negative control. (c) Clones 1 – 12 were analysed on Ganciclovir sensitivity (5 µg/ml) and resistance against Puromycin (5 µg/ml). Growth was analysed by methylene blue staining in the tissue culture plates. In conclusion, clones 4 and 9 still hold the PuroTK marker cassette from one of the LPV integrates, despite being $RFP^{+}/GFP^{-}$.

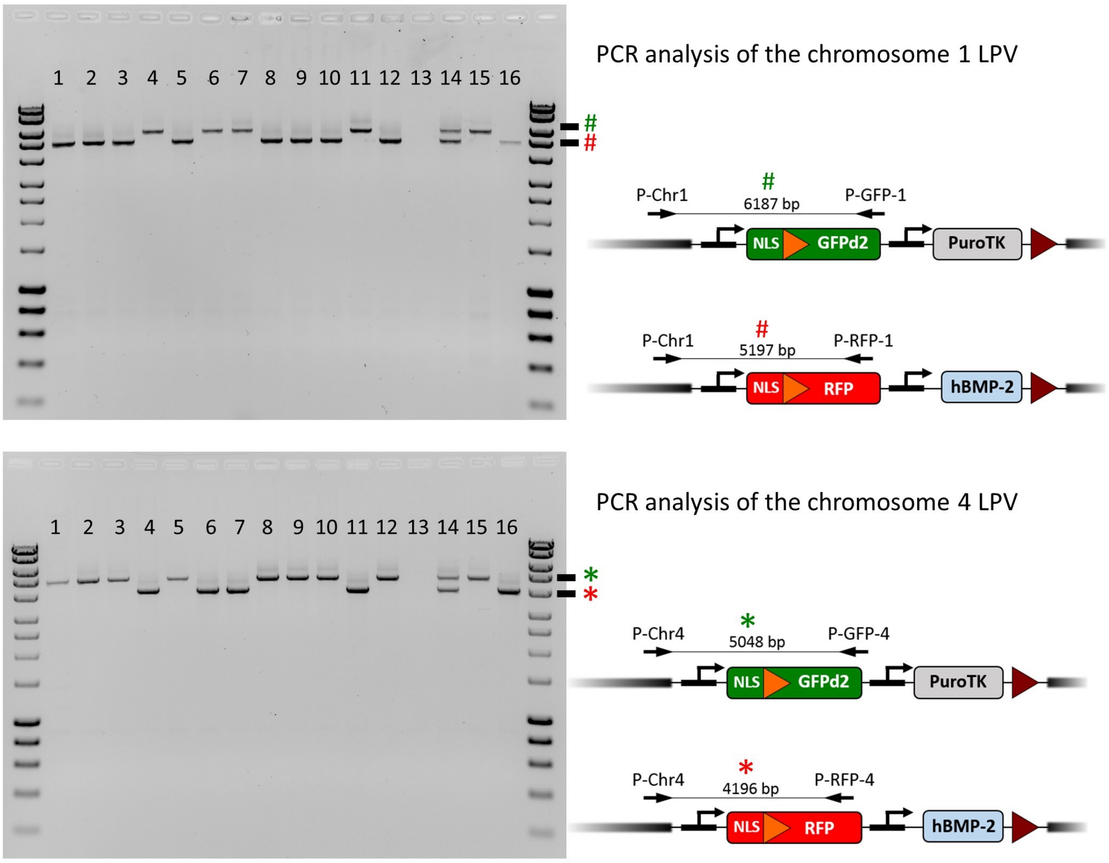


1-12: GFP+/RFP+ CHO.S18 clones 1-12
13: CHO wt
14: CHO.S18 RMCE sorted GFP+/RFP+ pool
15: CHO.S18
16: CHO.S18-R

**Supplemental Figure S5: Analysis of RMCE efficiency of individual LPV loci in CHO.S18**

Genomic DNA of 12 $GFP^+/RFP^+$ clones (lane 1 -12), the originating CHO.S18 RMCE sorted $GFP^+/RFP^+$ pool (lane 14), the parental CHOS18 clone (lane 15) and a cell sorted $GFP^-/RFP^+$ cell pool after RMCE (CHO-S18-R; lane 16) was analysed by two PCR reactions (one for each chromosomal locus) using three primers as indicated on the scheme to the right. PCR primers are specified in the supplemental table. Genomic DNA of CHO wt cells served as a negative control (lane 13). PCR products were analysed on agarose gels using Hyperladder 1kb as a size marker.

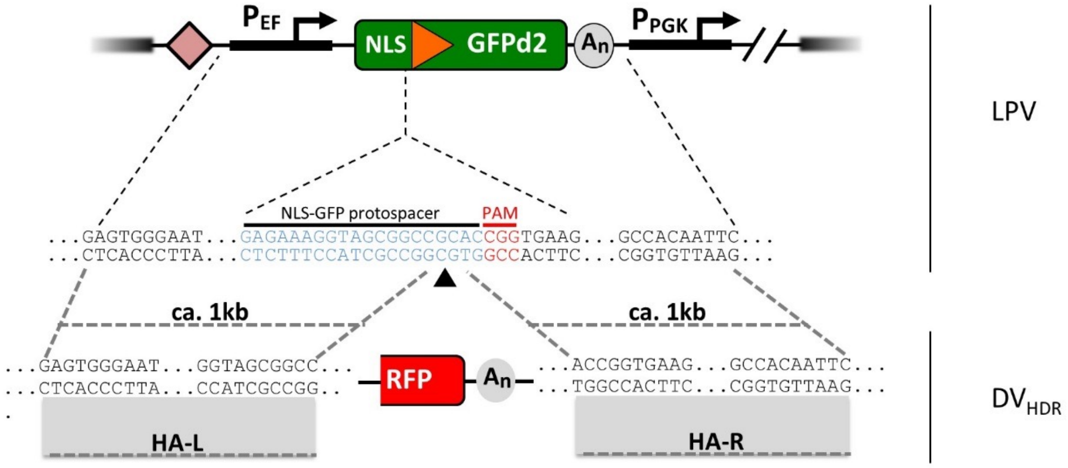


**Supplemental Figure S6: Protospacer and PAM sequence within the LPV and area of homology for gene targeting**

The PAM (red) and the protospacer (blue) sequence and the expected Cas9 cut site (filled triangle) are highlighted. The area of homology begins 1 bp away from the cutting site; homology arms are approximately 1 kbp in size.

Clone 2

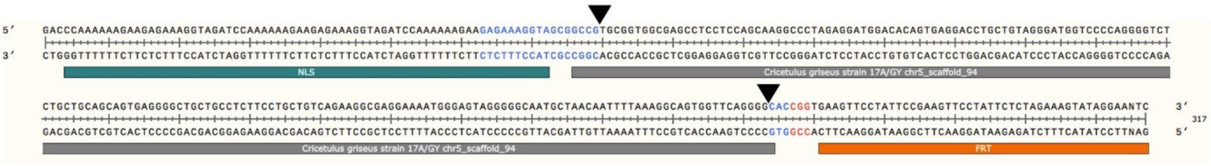


Clone 8

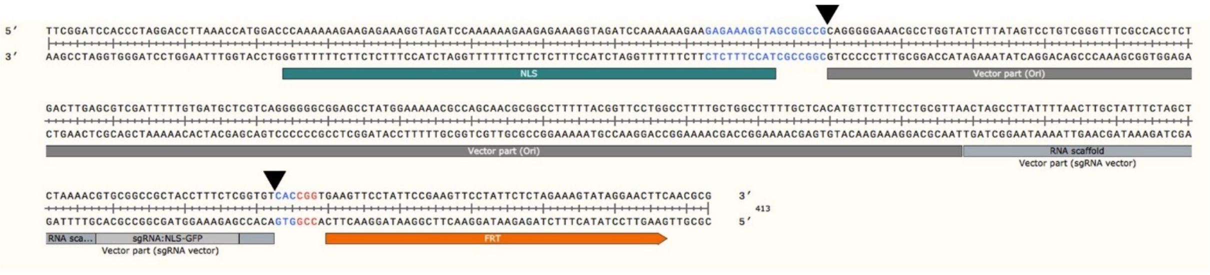


**Supplemental Figure S7: Irregular integration events in gene targeting via HDR in CHO.S18**

PCR analysis of HDR events in 12 clones indicated an irregular recombination event in two cases (see Fig. 5). Sanger sequencing of PCR products in question revealed integration of genomic part of the host cell (clone 2) and a vector fragment (i.e. bacterial origin of replication; present in both the $DV_{HDR}$ and the sgRNA/Cas9 expression plasmid; the sgRNA with part of the RNA scaffold sequence from the sgRNA vector; clone 8), respectively. In both cases, the integration occurred at the NLS-GFP-gRNA induced double strand break site. The PAM (red) and the protospacer (blue) sequence and the expected Cas9 cut site (filled triangle) are highlighted.

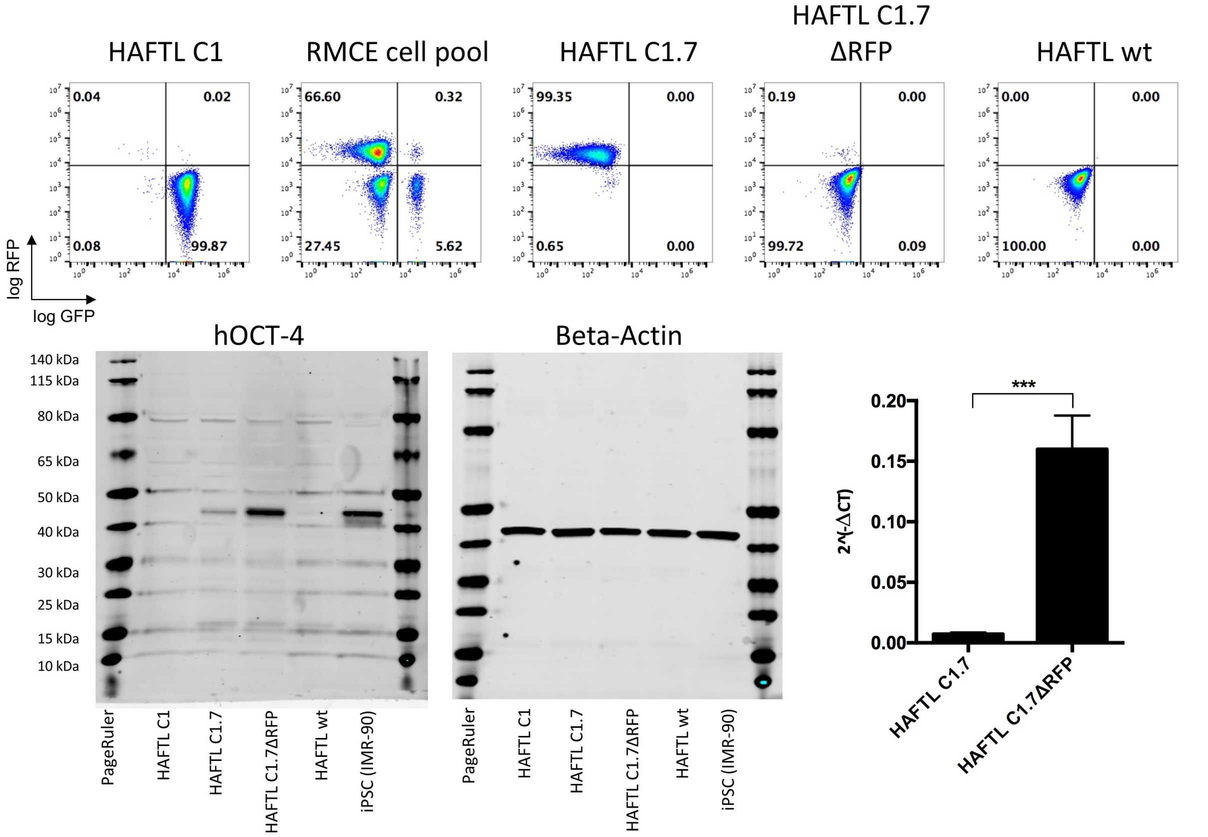


**Supplemental Figure S8: Stable RMCE experiments in HAFTL cells**

The LPV was integrated into HAFTL cells by lentiviral transduction (MOI 0.1). Cells were exposed to 3 µg/ml of puromycin for 1 week post transduction and the cell pool was used for subcloning by limiting dilution. The selected RMCE target clone HAFTL C1 was co-transfected with $DV_{RMCE}$(hOCT-4) and pCAAGS-Flpe-puro and exposed to 10 µg/ml Ganciclovir for selection of RMCE events (RMCE cell pool). The pool was used for subcloning by limiting dilution, resulting in HAFLT C1.7, and subsequently transfected with Cre plasmid for excision of the RFP marker. A flow cytometry sort for RFP- cells resulted in the HAFTL C1.7ΔRFP cell pool. hOCT-4 expression could be detected by immunoblot anaylsis and qPCR revealed a 22-fold upregulation in hOCT-4 expression after Cre-mediated RFP excision (n=3; P-value by unpaired t-test, ***p=0.0007). The uncropped blot pictures shown in S11f.

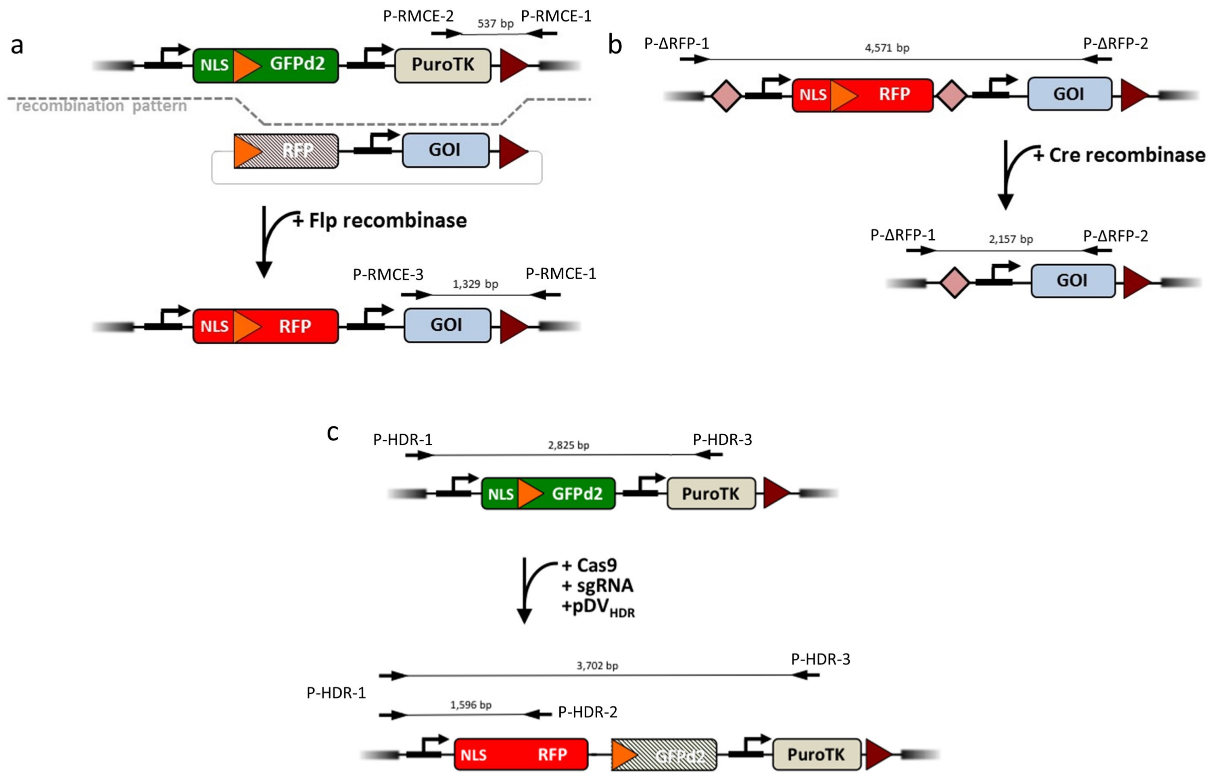


**Supplemental Figure S9: Schematic overview of PCR analysis of RMCE and HDR events**

Overview of recombination strategies and their PCR characterisation. **(a)** RMCE authenticity and integrity of recombination was checked with a multiplex PCR approach (see supplement table 1 for primer sequences). Here, three primers were used simultaneously. Upon accurate RMCE at all targets, the PCR will result in a single amplicon of 1,329 bp in size. P-RMCE-1 does not anneal to $DV_{RMCE}$. **(b)** RFP excision PCR is set over the RFP marker and its EF1α promoter sequence. The Cre-mediated excision reduces the amplicon size from 4,571 bp to 2,157 bp. **(c)** Integration of the RFP marker via the HDR pathway was characterised with two independent PCRs. The first PCR was set over the left homology arm of $DV_{HDR}$ (1,596 bp, P-HDR-1 and P-HDR-2; P-HDR-1 does not anneal to $DV_{HDR}$). This PCR product is indicative for site specific integration at the targeted site. The second PCR is set over the entire integrated RFP marker sequence. Here, successful gene targeting via the HDR pathway is indicated by an amplicon of 3,702 bp in size, whereas the parental locus will yield in a PCR product of 2,825 bp.

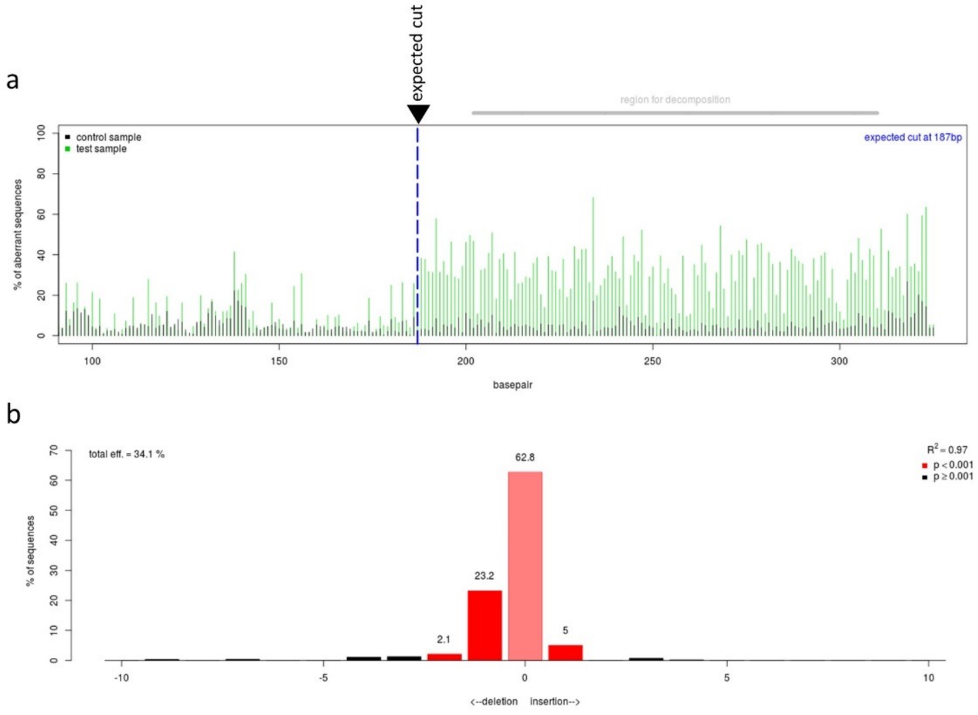


**Supplemental Figure S10: Indel quantification via TIDE analysis**

For quantification of indels, the purified PCR products were Sanger-sequenced and each sequence chromatogram was analysed with the *Tracking of In/dels by Decomposition* (TIDE) web tool (http://tide.nki.nl; Brinkman, E. K., Chen, T., Amendola, M. & van Steensel, B. Easy quantitative assessment of genome editing by sequence trace decomposition. *Nucleic Acids Res* **42**, e168 (2014)). **(a)** Visualizing of aberrant sequence signal and quality control of the sequencing data. TIDE aligns the sequence of the experimental sample with the control sequence and the guide sequence with expected cutting site is set by the users input (here 5'-GAGAAAGGTAGCGGCCGCAC-3'). TIDE then sets the region of decomposition (grey bar) for the quality control of the sequencing data. A high percentage of the aberrant sequence signal within the region of decomposition represents a high quality of the sequencing data. Aberrant sequence signal of the sample transfected with pX330-sgRNA:NLS-GFP (test sample) are shown in green, control sample without any further treatment is shown in black. **(b)** The decomposition at the cutting site yields the spectrum of indels and their frequencies. TIDE calculates deletion and insertion events by shifting the peaks of the sample and control sequence by 10 nucleotides to the left and to the right side of the cutting site. Here, the overall indel formation frequency is 34.1%. 23.2% are deletion events of 1 bp and 2.1% of 2 bp, while 5% are insertion events of 1 bp. $R^2$ is calculated by TIDE to assess the goodness of t.

**Supplemental Figure S11: Uncropped pictures of agarose gels and immunoblots as partially incorporated in the figures of the manuscript**

S11a: unedited gel pictures used in Fig 2b

S11b: unedited blot pictures used in Fig 3a

S11c: unedited gel pictures used in Fig 4b

S11d: unedited gel pictures used in Fig 5b

S11e: unedited gel pictures used in Fig S4b

S11f: unedited blot pictures used in Fig S5

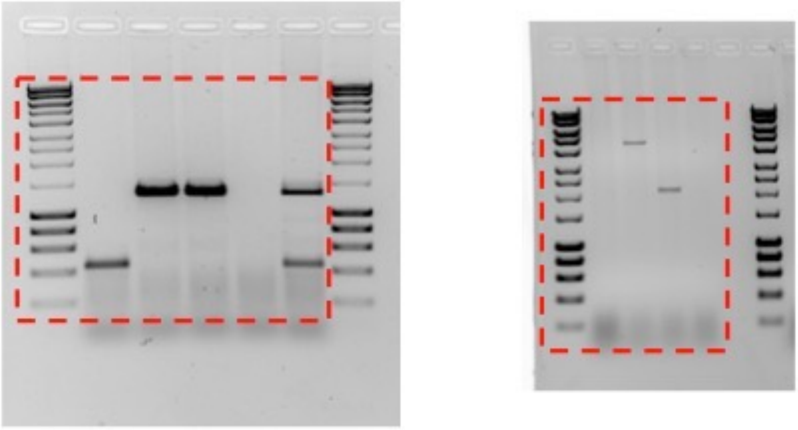

Suppl. Fig. S11a: unedited gel pictures used in Fig 2b

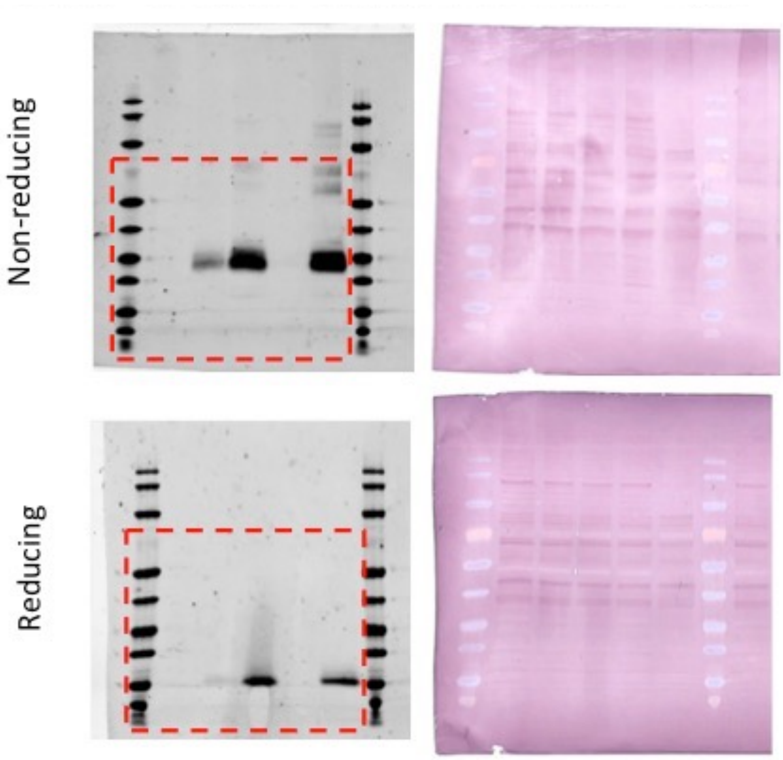


Suppl. Fig. S11b: unedited blot pictures used in Fig 3a

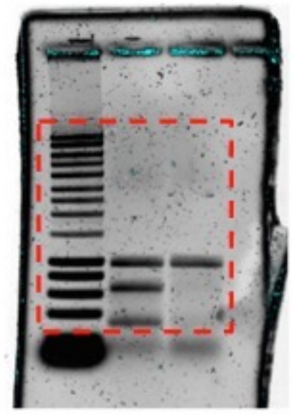

Suppl. Fig. S11c: unedited gel pictures used in Fig 4b

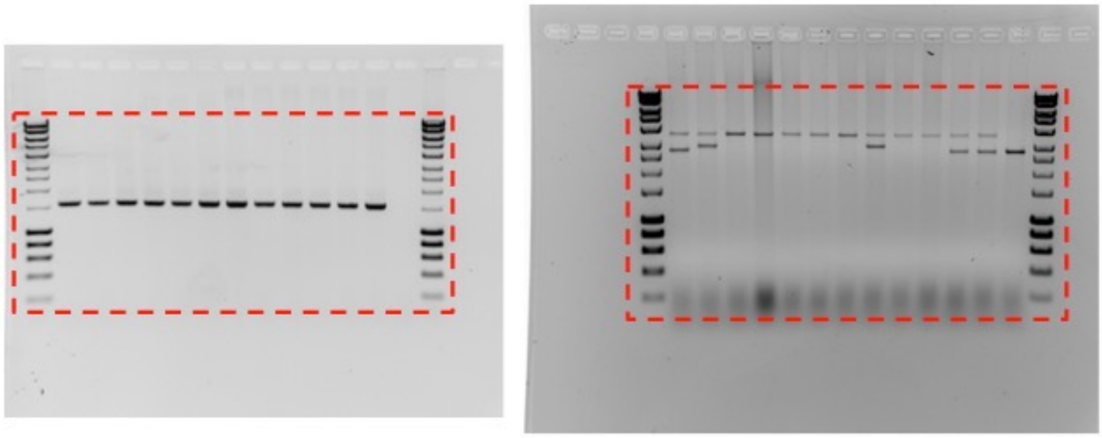

Suppl. Fig. S11d: unedited gel pictures used in Fig 5b

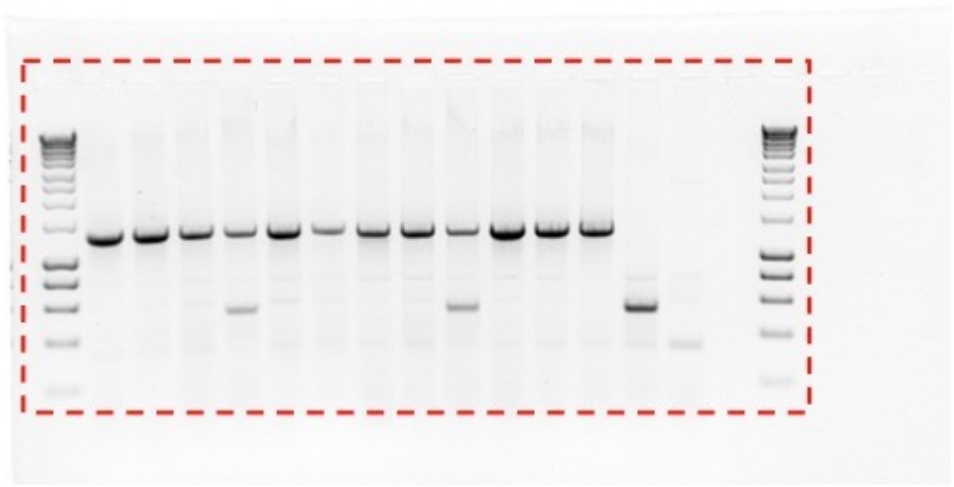

Suppl. Fig. S11e: unedited gel pictures used in suppl. Fig S4b

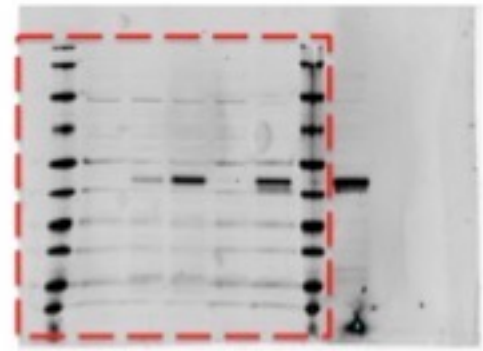

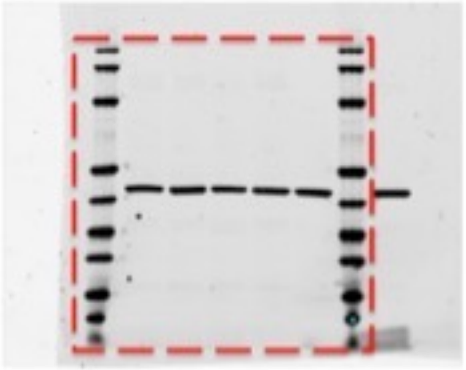

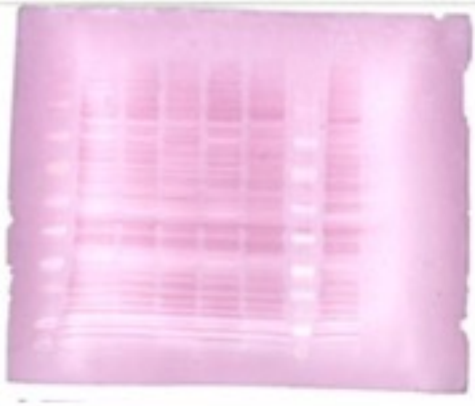

Suppl. Fig. S11f: unedited blot pictures used in suppl. Fig S8

**Supplemental Table 1: List of oligonucleotides**

Oligonucleotides used as adapters for NGS hold two additional modifications. 5’end is phosphorylated (P) for ligation purposes and 3’end is blocked with an amine group (N) to prevent extension of the 3’end during PCR.

| Purpose | Oligoname | Sequence (5'-3') |
|---|---|---|
| **PCR analysis** | | |
| | | |
| HDR sort clone PCR analysis | P-HDR-1 | CATGATCTCGTGAAGCGAGC |
| | P-HDR-2 | CAGCTTCATGTGCATGTTCTCC |
| | P-HDR-3 | TCGATGTTGTGGCGGATCTT |
| | | |
| | | |
| ΔRFP PCR analysis | P-ΔRFP-1 | TGGAACCGGAACCCTTAAACAT |
| | P-ΔRFP-2 | GAATCCATGGTTGGCGTGTC |
| | | |
| | | |
| RMCE multiplex PCR analysis | P-RMCE-1 | TCCTGAGCGAGTTCGAGTTG |
| | P-RMCE-2 | AGGGACAGCAGAGATCCAGT |
| | P-RMCE-3 | TGGATTACGACCAATCGCCC |
| | | |
| | | |
| T7E1 assay | T7E1 sgRNA:NLS-GFP fw | AATCGGACGGGGGTAGTCTC |
| | T7E1 sgRNA:NLS-GFP rev | GGCGGACTTGAAGAAGTCGT |
| | | |
| | | |
| hOCT-4 qPCR analysis | RM GapdH fw | AAGGGCTCATGACCACAGTC |
| | RM GapdH rev | GGATGCAGGGATGATGTTCT |
| | hOCT4 fw | GAAGGATGTGGTCCGAGTGT |

| | | |
|---|---|---|
| | hOCT4 rev | GTGAAGTGAGGGCTCCCATA |
| | | |
| | | |
| RMCE multiplex Chr.1 and Chr.4 analysis | P-RFP-1 | GTGCCCTCCATGTACAGCTT |
| | P-Chr1 | CAGAAGCAGACCTCAGTGCT |
| | P-GFP-1 | TGGATGTGGAATGTGTGCGA |
| | P-RFP-4 | TCACCCCGTTGATCTTGACG |
| | P-Chr4 | CCTCCCAACAAACCATCCCA |
| | P-GFP-4 | TACCCGCTTCCATTGCTCAG |
| | | |
| | | |
| **Cloning** | | |
| | | |
| sgRNA | sgRNA:NLS-GFP upper | AAACGTGCGGCCGCTACCTTTCTC |
| | sgRNA:NLS-GFP lower | CACCGAGAAAGGTAGCGGCCGCAC |
| | | |
| | | |
| Homology arms for HDR | right homology arm fw | AAAACTTAAGACCGGTGAAGTTCCTATTCCGAAGTTCCTATTCTCTAGAAA |
| | right homology arm rev | AAAAGGTACCTAGAATTGTGGCGATCGCCACACAAAAAACCAACACACA |
| | left homology arm fw | AAAAAGATCTGAGTGGGAATTGGCTCCGGT |
| | left homology arm rev | AAAACTCGAGAAGGCCGCTACCTTTCTCTTCT |
| | | |
| | | |
| hBMP-2 | BamH1-hBMP2 forward | AAAGGATCCATAACCATGGTGGCCGGGAC |
| | hBMP2-Not1 reverse | GCGGCCGCTATCTAGCGACACCCACAACCCT |
| | | |
| | | |

| | | |
|---|---|---|
| Flpe-BFP | upper Flpe color | TAAGCGGCCGCAAACCACAACCGTGGACCGGTAAACTTAAGAAATCGCGACTGCA |
| | lower Flpe color | GTCGCGATTTCTTAAGTTTACCGGTCCACGGTTGTGGTTTGCGGCCGCTTAAT |
| | PCR BstX1-BFP GAT | AAACCACAACCATGGATAGCGAGCTGATTAAGGAGAACATG |
| | PCR BFP Nru1 | CCATAGAGCCCACCGCATCC |
| | | |
| | | |
| **Sequencing** | | |
| | | |
| Adapter for NGS | Ad upper | CTACACGACGCTCTTCCGATCTCGGGCTGC |
| | Ad BamH1 lower | P-GATCGCAGCCCG-N |
| | Ad Nhe1 lower | P-CTAGGCAGCCCG-N |
| | Ad Hind3 lower | P-AGCTGCAGCCCG-N |
| | | |
| | | |
| 1st PCR round NGS | ProVir_F | CAGACGTGTGCTCTTCCGATCTAGCCGCCTAGCATTTCATC |
| | ProVir_R | CAGACGTGTGCTCTTCCGATCTTTCGCTTTCAAGTCCCTGTT |
| | Ad1-f | CTACACGACGCTCTTCCGATCT |
| | | |
| | | |
| 2nd PCR round NGS (Index) | TS-PCR-f | AATGATACGGCGACCACCGAGATCTACACTCTTTCCCTACACGAC |
| | TS-Idx44 | CAAGCAGAAGACGGCATACGAGATATTATAGTGACTGGAGTTCAGACGTGTGCTCTTC |
| | TS-Idx45 | CAAGCAGAAGACGGCATACGAGATGAATGAGTGACTGGAGTTCAGACGTGTGCTCTTC |
| | | |
| | | |
| CHO.S18 5'end sequencing | CHO.S18 chrom1 5 end fw | AGGGCCTGCTCCTTAGAGAA |

| | | |
|---|---|---|
| | CHO.S18 chrom1 5 end rev | GACGGGCACACACTACTTGA |
| | | |
| | | |
| | CHO.S18 chrom1 5 end fw | TGGCATCCCATCCACAAACA |
| | CHO.S18 chrom1 5 end rev | GACGGGCACACACTACTTGA |
| | | |
| | | |
| Sanger Sequencing (Seqlab) | PEN1-737R | TCCAGCTCGACCAGGAT |
